# Hippocampal–Prefrontal Communication Subspaces Align with Behavioral and Network Patterns in a Spatial Memory Task

**DOI:** 10.1523/ENEURO.0336-24.2025

**Published:** 2025-09-18

**Authors:** Ryan A. Young, Justin D. Shin, Ziyi Guo, Shantanu P. Jadhav

**Affiliations:** ^1^Neuroscience Program, Department of Psychology, Brandeis University, Waltham, Massachusetts 02453; ^2^Undergraduate Program in Neuroscience, Brandeis University, Waltham, Massachusetts 02453

**Keywords:** canonical correlation analysis, communication subspace, hippocampal–prefrontal interactions, memory-guided behavior, sharp-wave ripples, theta rhythms

## Abstract

Rhythmic network states have been theorized to facilitate communication between brain regions, but how these oscillations influence communication subspaces, i.e., the low-dimensional neural activity patterns that mediate interregional communication, and in turn how subspaces impact behavior remain unclear. Using a spatial memory task in rats (male Long–Evans rats), we simultaneously recorded ensembles from hippocampal CA1 and the prefrontal cortex (PFC) to address this question. We found that task behaviors best aligned with low-dimensional, shared subspaces between these regions rather than local activity in either region. Critically, both network oscillations and speed modulated the structure and performance of this communication subspace. To understand the communication space, we visualized shared CA1–PFC communication geometry using manifold techniques and found ring-like structures. We hypothesize that these shared activity manifolds are utilized to mediate the task behavior. These findings suggest that memory-guided behaviors are driven by shared CA1–PFC interactions that are dynamically modulated by oscillatory states, offering a novel perspective on the interplay between rhythms and behaviorally relevant neural communication.

## Significance Statement

This study reveals that shared communication subspaces between the hippocampus and prefrontal cortex are aligned with both behavioral patterns and network oscillations during a spatial memory task. We demonstrate that these shared subspaces robustly predict task behavior, while local activity in either region alone does not. The organization of these task subspaces into differing manifolds demonstrates task information in interregional coordination, thought to be critical for memory-guided behavior. Moreover, our findings highlight the significance of theta power in modulating these communication dynamics. These insights provide a deeper understanding of the interregional neural mechanisms underlying mnemonic and behavioral processes, which are of broad interest to the neuroscience community.

## Introduction

Cognitive functions, including memory and decision-making, hinge on the coordinated interaction across multiple brain regions. In the hippocampus, such coordination prominently anchors to hippocampal oscillatory rhythms (Colgin, 2016). These rhythms have been hypothesized to help synchronize downstream target networks by aligning periods of excitability (McKenzie, 2018). Parallel to this idea, other communication hypotheses have been put forth to explain tightly regulated neuronal communication across regions, including communication subspaces (Kohn et al., 2020; Semedo et al., 2020) and communication through coherence (Fries, 2015). Yet it remains unclear how these seemingly parallel aspects of communication—rhythms and alignment of firing to communication subspaces—could relate to one another.

Hippocampal activity in rodents during behavior and sleep displays a diverse cast of high- and low-frequency events. At the high-frequency end, sharp-wave ripples (SPW-Rs) constituting some of the most synchronous events in the brain occur during periods of immobility and during non-REM sleep (Buzsáki, 2015; Zhang et al., 2021). These transient high-frequency bursts have been implicated in interregional memory consolidation and retrieval (Battaglia et al., 2011). During quiescent periods, SPW-Rs are known to mediate interactions with extrahippocampal areas, including the prefrontal cortex (PFC) and subcortical structures (Kaplan et al., 2016; Karimi Abadchi et al., 2020; Norman et al., 2021; Nitzan et al., 2022), comprising key times of inter- and intra-areal messaging. In contrast, lower frequencies, in particular theta rhythms, predominate during active exploration, motor behavior (Joshi et al., 2022), and memory processing (Shimbo et al., 2021; Seger et al., 2023). Theta oscillations and theta phase-modulated neuronal activity have been causally linked to memory encoding and retrieval (Siegle and Wilson, 2014). Theta may therefore enable and invoke these separate processes in part through regimented and unique timing of interareal inputs and outputs within each theta wave (Tsodyks et al., 1996; Bezaire et al., 2016; Fernández-Ruiz et al., 2019; Mysin, 2023), possibly linked to gamma rhythms (Colgin et al., 2009; Fernández-Ruiz et al., 2017). Hippocampal spiking thus is known to interact with oscillation timing and vice versa.

Apart from oscillations, communication fundamentally also arises from structural possibilities available in the network, giving rise to only limited manifolds that do not span all possible firing configurations. Studies using recurrent networks have provided valuable insights into communication manifolds. Sparse connectivity can create low-dimensional dynamics (Herbert and Ostojic, 2022), and coupling groups of such recurrent networks through specific internetwork projections create even lower-dimensional structures, a low-rank manifold where activity passes between networks (Barbosa et al., 2023; Kozachkov et al., 2022). These narrow-dimension dynamics confer advantages like enhanced stability and prescribe a limited range of spiking patterns when pairs of networks engage in communication (Herbert and Ostojic, 2022).

A critical insight is that linear subspaces can approximate variations on nonlinear manifolds, assuming the space is not overly curved (Johnson et al., 2003; Gallego et al., 2017; Semedo et al., 2019). Using such a linear approximation, Semedo et al. (2019) made important discoveries about visual cortical communication, namely, interareal V1–V2 interactions occurred in a lower dimension than intra-V1 communication. This V1–V2 subspace was consistent across stimuli, unaffected by nonlinearity, and distinct from part of V1's subspace for private V1-to-V1 messaging (Semedo et al., 2019; Iyer et al., 2021). These principles have been substantiated and expanded in other work. For instance, Srinath et al. (2021) found cortical communication geometry was largely stable when attention shifted, though information flow increased along it, and MacDowell et al. (2025) later expanded the investigation to subserve an area's multiple possible downstream targets. With large-scale recording, they discovered subspaces projecting to a region's targets were often multiplexed and overlapping. Thus, as suggested by models (Barbosa et al., 2023), a population can send to or receive from multiple targets and sources by aligning spiking activity to the appropriate subspace (Semedo et al., 2019; MacDowell et al., 2025).

These two forms of communication, rhythmic coordination and subspace alignment, have been well described. However, whether and how these two communication modes fit together is not clear and remains an important question (Esparza et al., 2023). Are they separate mechanisms or intertwined, different perspectives on a shared process? In 2023, Kim et al. observed one possible interaction. In a motor learning task, they found prefrontal (PFC)–motor cortical (M1) communication-subspace-aligned activity intensifies when M1 slow oscillations (SO) follow hippocampal SPW-Rs. In other words, complexes of hippocampal SPW-R and cortical SO mark time periods where PFC and M1 share activity along their communication space. This exemplifies a possible rhythmic event entangling communication spaces. Theoretically, neural manifolds can exhibit limit cycles and rotations in spiking, which could evolve within or between communication and private subspaces or be completely separate. However, direct insights remain scarce. Lastly, communication subspaces have been characterized in response to perceptual stimuli but remain unexplored for cognitive and task-related behaviors.

Since hippocampal activity patterns exemplify rhythmic communication and with evidence that the task under study depends on hippocampal–prefrontal communication (Maharjan et al., 2018), we decided to address these gaps by investigating the following questions: are hippocampal–prefrontal communication subspaces related to task behavior and how do rhythmic network patterns sculpt them? We also asked if these relationships evolve with learning. Clarifying the linkage between rhythms and subspaces could reveal how neural populations temporally harmonize and multiplex communication during cognitive processing.

## Materials and Methods

### Subjects

Seven adult male Long–Evans rats served as subjects in this study. Their weights ranged from 450 to 550 g at the ages of 4–6 months. The Institutional Animal Care and Use Committee at Brandeis University approved all procedures, which followed the US National Institutes of Health guidelines. Every animal was housed individually and maintained on a 12 h light/dark cycle.

### Animal pretraining

The animals were habituated through daily handling for several weeks prior to training. After habituation finished, the rats underwent food deprivation to reach 85–90% of their *ad libitum* weight. The rats were then pretrained to run along a linear track (∼1 m long) to receive rewards of sweetened evaporated milk. They also became accustomed to spending time in a high-walled, opaque sleep box (∼30 × 30 cm). Following linear track pretraining, the animals received surgical implantation of a multitetrode drive, with local field potentials (LFPs) measured relative to a cerebellar ground screw. Electrodes were not moved for at least 4 h pre- and during recording (Tang et al., 2017; Shin et al., 2019; Tang et al., 2021).

### Microdrive implantation

Six animals of seven animals were implanted with a multitetrode drive containing 32 independently moveable tetrodes targeting the right dorsal hippocampal region CA1 (−3.6 mm AP and 2.2 mm ML) and right PFC (+3.0 mm AP and 0.7 mm ML; 16 tetrodes in CA1 and 16 in PFC for four animals; 13 in CA1 and 19 in PFC for two animals). One animal was implanted with a multitetrode drive containing 64 independently moveable tetrodes targeting the bilateral CA1 of dorsal hippocampus (−3.6 mm AP and ±2.2 mm ML) and PFC (+3.0 mm AP and ±0.7 mm ML; 30 tetrodes in CA1 and 34 tetrodes in PFC). On the days following surgery, hippocampal tetrodes were gradually advanced to the desired depths with characteristic EEG patterns (sharp-wave polarity, theta modulation) and neural firing patterns as previously described (anon). One tetrode in the corpus callosum served as a hippocampal reference (CA1 REF), and another tetrode in overlying cortical regions with no spiking signal served as a prefrontal reference (PFC REF). The reference tetrodes reported a voltage relative to a ground (GND) screw installed in the skull overlying the cerebellum. Electrodes were not moved at least 4 h before and during the recording day (anon).

### Behavioral training

Following recovery from surgical implantation (∼7–8 d), the animals were food-deprived again and pretrained on a linear track for at least 2 d. Then the novel W-track sessions began (Fig. 6*A*; ∼80 × 80 cm with ∼7-cm-wide tracks). The rats were introduced to the W-track for the first time and learned the task rules over eight behavioral epochs on a single day. Each 15–20 min epoch was followed by a 20–30 min rest session in the sleep box (total recording time, ∼6 h). As depicted in Figure 6*A*, the rats performed a hippocampus- and prefrontal-dependent continuous alternation task on the W-maze in the manner of Zielinski et al. (2019). The rats had to alternate their visits between the left (L), right (R), and center (C) wells to receive automated rewards upon crossing infrared beams at the reward sites. The four correct trajectory types were C-to-L, L-to-C, C-to-R, and R-to-C. Learning performance was estimated via a state-space model as described in Jadhav et al. (2012).

### Electrophysiological recording

The hippocampal and PFC activities were recorded using 30–64 tetrodes positioned in the right dorsal hippocampal CA1 region and PFC. Recording sites were verified via histology. The individually movable tetrodes targeted the desired regions. Data were collected with a SpikeGadgets acquisition system. Spike signals were bandpass filtered 600 Hz to 6 kHz and sampled at 30 kHz. LFPs were bandpass filtered 0.5 Hz to 400 Hz and sampled at 1.5 kHz. The overhead color CCD camera tracked animal's position and running speed via affixed LEDs (30 fps). Single units were identified through manual clustering based on amplitude, principal components, and spike width using the MatClust software (M.P. Karlsson). Only well-isolated neurons with stable waveforms were included as in Shin et al. (2023). Neurons firing below 0.1 spikes/s on average were excluded across all analyses.

### LFP preprocessing and coherence quantification

The LFP underwent bandpass filtering in the delta (0.5–4 Hz), theta (6–12 Hz), and ripple (150–250 Hz) bands. Zero-phase infinite impulse response Butterworth filters were utilized. Coherence calculations were performed with the Chronux (http://chronux.org/, RRID:SCR_005547) MATLAB software package for spectral analysis.

### Rank regression analysis

#### Partitioning

Spikes were counted in 50 ms bins across the run recording periods. To examine related activity between the two areas, we reasoned that fluctuations in hippocampal neurons could relate to fluctuations in PFC neurons and vice versa.

The hippocampal and prefrontal neurons were matched by their average firing rates. The distribution of firing rates was divided into 20 equal-range bins. The minimum number of neurons from each bin was randomly selected from both areas. Since more hippocampal cells were typically recorded, the distribution of selected target cells matched the prefrontal distribution. The target population size ranged from 11 to 65 units (mean, 29.2). Partitioning was repeated 50 times, selecting different sets of source neurons and mean-matched target neurons each time. The partitions were consistent across different methods of generating windows of interest (described below) to control dimensionality analyses. Each source and target pair underwent reduced-rank regression (RRR; Semedo et al., 2019).

#### Windowing of rhythmic periods

High LFP activity windows were selected when the oscillation of interest exceeded 85% of the band power, using a 300 ms window beginning at the threshold trigger of the oscillation. Low oscillatory activity windows occurred when power fell below 15% of the band (*SFig. Window Partitions*). Overlapping windows were removed, both within and between different activity patterns. The windows were matched so that all oscillation patterns and strengths had approximately uniform windows across the recording period. Statistical tests based on comparisons of window states (high/low, intra-/interareal, rhythmic band) were corrected by Benjamini–Hochberg for multiple comparisons.

#### Rank regression

RRR asks whether a mapping from source population X to a target population Y can be reduced to lower-dimensional structure.

We first correlated spiking activities between source and target regions using linear regression:

Y=XB+ε.

X represents the p source neurons by T timepoints matrix, where T timepoints come from N windows each containing $t_{w}$ timepoints. Y represents the T timepoints by q target neurons matrix. The coefficient matrix B ( p×q) combines activity in each row of X to minimize the squared error, and ε is i.i.d. Gaussian noise.

The OLS estimate of B is as follows:

$$B_{\mathrm{OLS}}=(X^{T}X{)}^{-1}X^{T}Y.$$

Note that this is standard ordinary least squares regression. We then apply RRR to this OLS solution to identify lower-dimensional structure.

To test whether a subspace of the source neurons could still predict the target activities, we applied RRR. This gradually took the first m principal components of $B_{\mathrm{OLS}}$ to reduce its rank to $B_{\mathrm{RRR}}$:

$$B_{\mathrm{RRR}}^{\left(m\right)}=B_{\mathrm{OLS}}V_{1:m}V_{1:m}^{T}.$$

For each m we predicted the following:

$${\hat{Y}}_{\mathrm{test}\;}^{\left(m\right)}=\;X_{\mathrm{test}}B_{\mathrm{RRR}}^{\left(m\right)},$$

computing a cross-validated coefficient of determination $R^{2}(m)$.

The optimal rank $\hat{m}\;$ was the smallest m whose mean $R^{2}(m)$ lay within one standard error of the full-rank model, yielding the minimal set of source-population modes required to account for the target activity.

### Canonical correlation analysis (CCA)

CCA was used to identify correlated activity patterns between the hippocampal CA1 and PFC populations. CCA finds linear combinations of the two populations that maximize correlation, known as canonical variates (Hotelling, 1936; Steinmetz et al., 2019). Dimensions resulting from CCA analysis form communication subspaces, and private subspaces are orthogonal to communication subspace dimensions. In particular, we used CCA to continuously sample the overall communication subspace in a temporally resolved manner.

Spiking activity was binned in 50 ms windows. CCA was applied to the binned spike count matrices of the CA1 and PFC populations to derive canonical variates that capture maximally correlated activity between the regions. Fivefold cross-validation was used to generate robust canonical variates (Steinmetz et al., 2019).

For examining individual canonical variates (Figs. 2, 6), the top three variates explaining the most correlated activity were analyzed. For behavioral prediction analyses (Fig. 5), the top 10 canonical variates were used, below the typical optimal communication subspace dimension found through other analyses (Fig. 4). Figure 4 predicted the first CCA component using network patterns or speed.

For each canonical variate dimension, CCA produces a temporal activity pattern for CA1 (*U*) and PFC (*V*). The U and V temporal patterns were used to calculate aligned activity along the U=V unity line as (U+V)/sqrt(2), capturing reciprocated activity between areas. Orthogonal activity was calculated as (U−V)/sqrt(2) to measure unreciprocated activity.

### Behavioral prediction with linear regression

Linear regression models were trained to predict rat behaviors from hippocampal–prefrontal communication space activity patterns derived with CCA. Models were constructed using TuringGLM.jl, a Julia package for Markov chain Monte Carlo (MCMC) sampled generalized linear models (GLMs; Storopoli et al., 2021). For each animal and behavior, 2,000 MCMC samples were generated per GLM using the No U-Turn Sampler algorithm (Betancourt, 2018).

The 50,000 random samples were used for training, and the remaining were held-out for testing, with separate GLMs per animal and behavior. Bernoulli regression was used for binary behavioral variables. Robust *T*-distributed regression was used for continuous variables to account for outliers.

The CCA source U and target V temporal activity patterns for the top 10 canonical variates were used as input features. These were projected onto an orthonormal aligned (reciprocated) and orthogonal aligned (unreciprocated) basis for the regression.

The models predicted velocity, acceleration, linearized position (0, center; 1, side wells), correct versus error trials, outbound versus inbound trajectories, integrated heading direction (IdPhi), and left versus right trajectories.

For Figure 5*A*–*F*, these models were computed using an entire session (day) of data. All animals with stable convergence of MCMC chains in all epochs were analyzed (*N* = 6 animals out of 7). Epoch-wise models were created for Figure 5*G*, for which *N* = 7 animals were used.

### Mixed behavior and LFP models

We created many different linear model specification formulas to test whether behavior, LFP power, or some combination thereof better drove communication subspace activity. The goal was to understand whether one could attribute shared activity to one variable alone or the other or if, instead, the activity requires both behavior and rhythmic network patterns.

In order to explore the information present in the parts versus the whole, we created data splits of animal, epoch, and each of three continuous behaviors (velocity, IdPhi, linear distance). In each split, we trained two models with either only behavior predicting the top shared activity component (R∼B) or theta local field power predicting the top shared activity component (R∼θ), and then we created five different models that incorporated some combination of these sources:

We assessed the model fit using metrics such as *R*-squared and AIC to ensure against overfitting for each model. We then compared the performance of these models to determine whether the inclusion of both behavior and theta power, as well as their interaction, provided a better fit to the data compared with models with only one predictor. This allowed us to assess the relative contributions of behavior and rhythmic network patterns in driving shared activity in the communication subspace and wholistic information beyond the parts indicated by higher $R^{2}$ with lower AIC score on combined models.

### Theta coherence over learning

To examine how theta coherence changed over learning, multiple coherence measures were calculated between CA1 and PFC as rats traversed the W-track across epochs. Coherence was quantified using both multitaper coherence and weighted phase lag index (WPLI). WPLI provides increased robustness to noise confounds.

For both coherence measures, normalization was performed in two ways: (1) *z*-scoring across time within frequency bands per animal and (2) min–max scaling per animal to a 0–1 range per frequency using scikit-learn applied to each animal's full dataset. Data were bootstrapped such that each animal contributed equal samples to a given bootstrap. These two procedures correct for animals differing in measurement magnitude and for imbalances in the number of temporal samples for animals.

To assess changes over learning, we sampled matched random subsets of times from each animal per epoch or spatial bin to ensure equal weighting across animals. Mean coherence and 95% confidence intervals were calculated from 1,000 resampled subsets.

Spatial binning examined coherence differences across track locations. Analyses also evaluated coherence changes over learning epochs.

### Communication–rhythm coupling over learning

To quantify the relationship between communication space activity and theta power/coherence over learning epochs, we trained linear regression models. The input features were aligned, and orthogonal temporal activity patterns were found in the first canonical variate. The output being predicted was either theta power or coherence. Standard linear regression was used to model the relationship and obtain descriptive statistics. One model was fitted per epoch to capture relationship changes over learning.

Model performance was summarized by the coefficient of determination $\left(R^{2}\right)$. To obtain a population estimate, 10 subsets were randomly sampled and modeled per animal. Mean $R^{2}$ and 95% confidence intervals were calculated across animals from these subsets to quantify how the linear relationship changed over epochs.

### Geometry of communication subspace

To investigate the relationship between neural activity and behavior, we constructed a pseudopopulation representation by pooling data across multiple animals. Spiking activity from simultaneously recorded hippocampal and prefrontal neurons was binned according to behavioral variables, including trajectory direction (inbound/outbound), turn direction (left/right), trajectory (of in–out/left–right), and linear distance along the track. For each unique combination of these behavioral variables, we calculated the mean firing rate of each neuron across all animals. We then ordered by relative trajectory within trajectory type (in–out/left–right) and concatenated neurons across animals. This resulted in a super animal firing rate matrix, where each row represented a unique behavioral state, and each column represented a neuron. We then performed CCA on the super animal data, separating the activity into hippocampal and prefrontal components. This allowed us to identify patterns of coactivation between the two brain regions and to visualize the shared and unique contributions of each region to the overall neural representation of behavior. The resulting CCA components were further analyzed and visualized using dimensionality reduction techniques to explore the underlying structure of the neural–behavioral space.

### Software and data analysis

Data processing and communication subspace measurements were performed using MATLAB (version 9.14, The MathWorks). Tidy data structures were visualized using Python (version 3.9, Python Software Foundation, https://www.python.org/) with the following libraries: Matplotlib (version 3.6; Hunter, 2007), Seaborn (version 0.12; Waskom, 2021), and Pandas (version 1.5.2; McKinney, 2010). MCMC methods were implemented in Julia (version 1.10) using the TuringGLM.jl packages previously described.

### Data and code availability statement

The data supporting the findings of this study are available in the Dandi Archive under the accession number DANDI ID#: 000978. The dataset includes all relevant recordings and metadata from the experiments described in this paper. The code used for analysis and generation of results in this study is available on GitHub at https://github.com/JadhavLab/CommSub and in the Extended Data. Detailed instructions for data processing and analysis are provided within the repository.

10.1523/ENEURO.0336-24.2025.d1 Extended Data 1 Download Extended Data 1, ZIP file.

## Results

**Figure 1.**
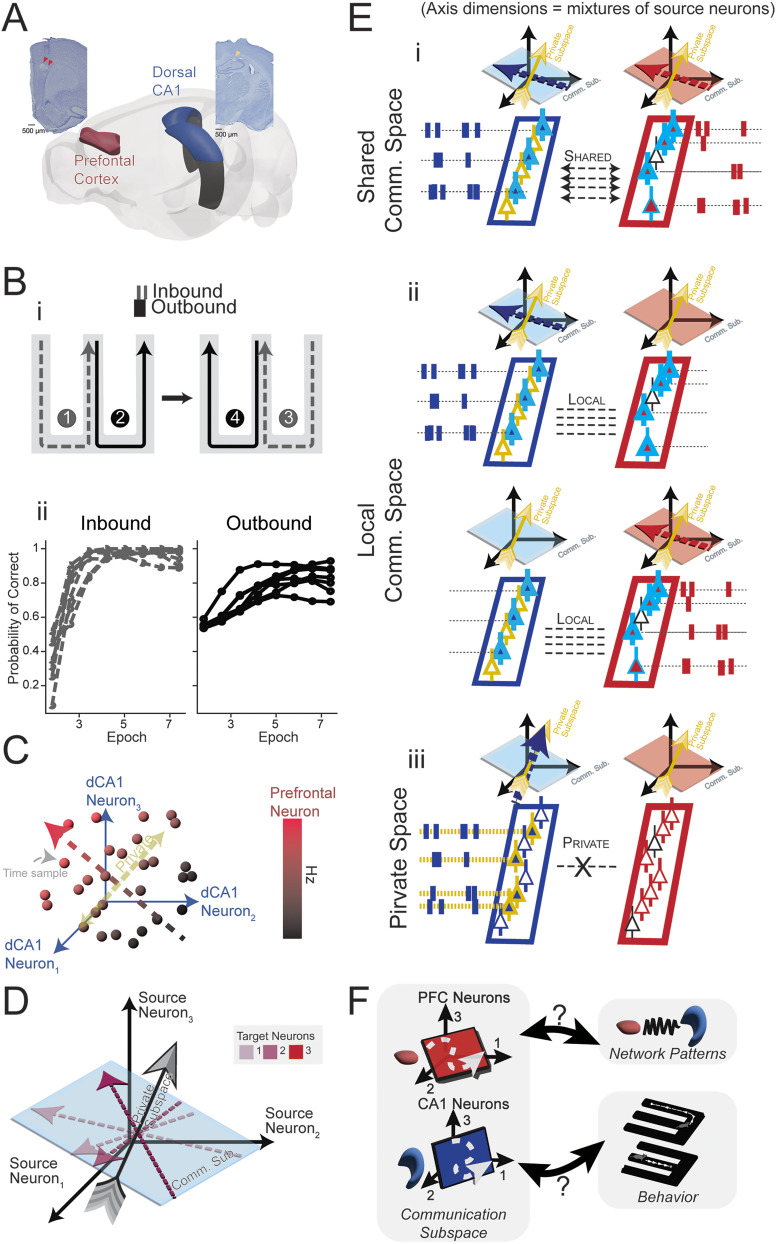
Connecting CA1–PFC communication subspaces to memory-guided behavior and network dynamics. ***A***, Schematic view of the targeted regions, PFC (highlighted in red) alongside dorsal CA1 (CA1, in blue). Insets display common electrode placements within the dorsal hippocampus (right) and PFC (left). ***B***, ***i***, Representation of the W-Track task learned over a single day, detailing the standard trajectory sequence numbered from 1 to 4. Dashed gray lines indicate inbound reference-dependent memory components, while outbound working memory phases are shown in solid black. During side arm visits, animals move to the center to receive a reward, whereas in the center arm, they recall their last visit and choose the alternate side. In panel ***ii***, we show inbound performance of animals *N* = 7 animals (left, gray dashed lines) and outbound performance (right, dark solid lines). ***C***, Demonstration of a one-dimensional communication subspace in a four-neuron system. Blue axes are formed by three CA1 source neurons. Scatter points display the firing correlation between the prefrontal target neuron and simultaneous CA1 neuron values. The bicolored (red/black) dotted arrow represents the direction in CA1 space that promotes increased activity in the target neuron—termed “communication space.” The yellow-dotted arrow indicates CA1 source directions with no impact on prefrontal firing denoted the “private space.” ***D***, Depiction of multidimensional communication within a three-neuron source and three-neuron target framework. Several target brain area neurons modulate activity spanning various dimensions of this subspace. “Private” dimensions orthogonal to communication subspace dimensions are those wherein alterations in source neuron activity do not cascade to downstream neurons. ***E***, ***i***, Schematic shows an example of shared communication subspace activity (Veuthey et al., 2020; Kim et al., 2023). Neurons which tend to cofire together across brain regions are depicted with a teal outline and neurons who do not statistically predict downstream neurons are depicted in yellow outline (private). When neurons who tend to cofire (communication space) actually do cofire, this is called “shared” activity. ***ii***, We call neurons which tend to cofire but fail to cofire in their typical activity ratio local activity (Kim et al., 2023)—activity that aligns to communication space in either CA1 or PFC, but not both. In this sense, activity is not appropriately mirrored in the other area—i.e., unreciprocated. ***iii***, The set of neurons which do not meaningfully add to extra-areal neuron firing rate prediction (Semedo et al., 2019). These neuronal configurations differ from local, in that they avoid neurons that commonly cofire between brain areas. We did not identify this fraction in the current study, but it can be obtained by collecting neurons in RRR that do not contribute information. ***F***, Graphical representation of the study’s central questions. Two depicted activity spaces from the PFC (in red) and CA1 (in blue) are aligned with network patterns and behavior. The schematic of the communication subspace highlights core inquiries: How effectively do these subspace activities signal task behavior and network patterns? And to what extent do specific network patterns, such as theta coherence and SPW-Rs, influence the manifestation of activity in these spaces?

Cognitive processes arise from interconnected brain areas working together. A notable example is memory-guided decision-making, a cognitive process shaped by the synchronized relationship between the hippocampal area CA1 and PFC. To probe this coordination, we simultaneously recorded CA1 and PFC activities as rats learned a spatial memory task on a continuous W-maze alternation task (Fig. 1*B*). The rats were taught to navigate varying paths on a W-maze across eight distinct training sessions interleaved with rest sessions within a single day (Fig. 1*B*) as in Shin et al. (2023). This single-day learned task structure permitted us to simultaneously monitor the activity of the same CA1 and PFC ensembles (target regions shown in Fig. 1*A*) throughout the course of learning. For success, this required rats to remember their most recent left/right choice while traversing from the central arm in order to choose the opposite side arm (outbound working memory task) and, subsequently, to return to the center arm to receive a reward (inbound; Fig. 1*Bii*). As rats learned and performed the task, we monitored the dynamics of cell firing and LFPs. This yielded 394 CA1 cells and 254 PFC neurons (Table 1) using our selection criteria (see Materials and Methods) across *N* = 7 animals for all analyses unless otherwise noted.

**Table 1.** Cell counts

Animal	CA1	PFC
1	60	30
2	44	26
3	44	16
4	39	21
5	41	48
6	34	38
7	132	75

Cell counts per animal within CA1 and PFC.

### Communication-aligned activity drives robust, repeatable behavior responses

Our first aim was to examine whether memory-guided behaviors in the task coordinate with communication-subspace-aligned activity. The fundamental question in communication subspaces is to find the changes in target brain area Y that arise with changes in a source area X. From the point of view of a linear model, this can be approximated with Y=XB. The instructive power of this technique lies in two observations. If we set $XB^{\prime }=Y=0$ and solve for $B^{\prime }$, the coefficients encode a special solution where source neuronal activity is insulated from influencing the target area *Y* (Kaufman et al., 2014); this is called a private space. Conversely, solving instead for XB=Y, the resulting remaining dimensions of the source neurons approximate the activity space of X that change Y, hence the shared communication subspace (schematic illustrating communication subspaces in Fig. 1*E*). This is illustrated in Figure 1*C* where we schematize a quad-neuron system with three CA1 source neurons and one PFC target neuron. An example of a one-dimensional communication subspace is shown via a line where source neuron activities comodulate with a target neuron (Semedo et al., 2019). Directions off from this axis and orthogonal to communication subspace are private: signals in the source area can fire without influencing the target, insulating it from communication between the networks. When many such target neurons are measured in this fashion (Fig. 1*D*), it comprises a collective communication subspace. We wished to understand how and if these spaces relate to memory-guided behavior, a hallmark of CA1–PFC network functions, and rhythmic network patterns (central questions illustrated in Fig. 1*F*), believed to be critical in CA1–PFC interaction (Floresco et al., 1997; Maharjan et al., 2018; Zielinski et al., 2020).

**Figure 2.**
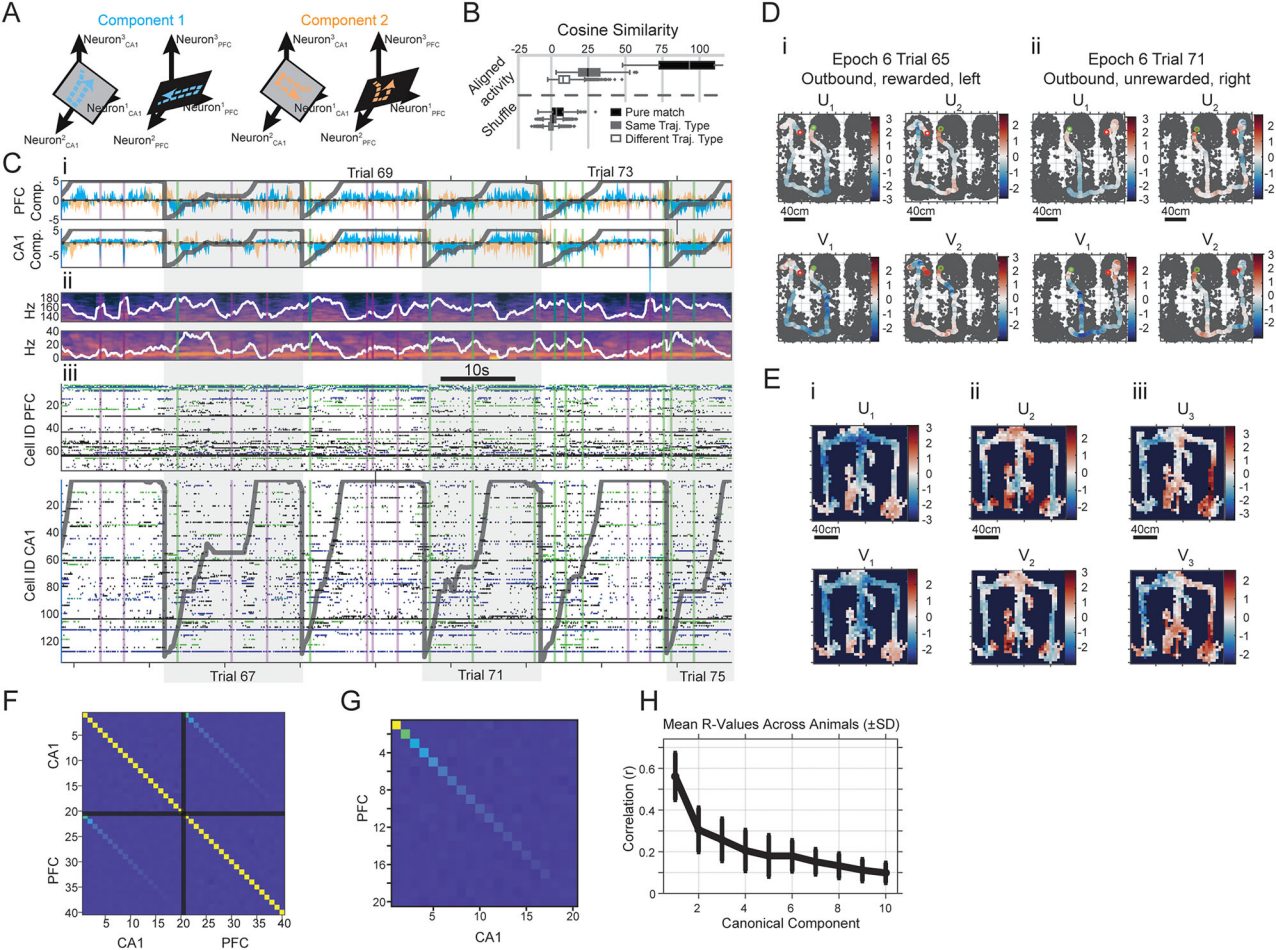
Canonical activity vectors in CA1 and PFC display consistent coordination with behavioral outcomes in animals. ***A***, Schematic representation of the first two subspace axes showing correlated neural activity in CA1 and PFC. Blue highlights the primary canonical variate of the communication subspace, while orange represents the second canonical variate activity axis. ***B***, The cosine similarity measurements indicate repeatability of canonical variate activity—how replicable canonical variate activity is across spatial trajectories. Each trajectory is binned into 100 locations, and 10 aligned canonical variates for the 100 locations are cosine compared with other trajectories. Measurements are split by trajectories. “Pure Match” indicates resampled points from the same exact trajectory (e.g., the 10th outbound-left trajectory); “Same Traj. Type,” trajectories that match type (e.g. compare all within inbound-left or all within outbound-right); or “Different Traj. Type,” trajectories that do not match type (compare inbound-left trajectories with inbound-right, etc.). Cosine measurements are performed with 100 dimensional vectors of mean UV activity per resampled trajectory. ***C***, ***i***, Illustration of neural activity patterns during multiple trials/trajectories along the principal correlated subspace dimensions (1 in blue, 2 in orange) for the hippocampal region (U) and PFC (V). Dark gray trajectories represent the animal’s linearized path during consecutive outbound trials. The neural activity patterns show qualitatively similar patterns across trajectories, illustrating repeatable communication structure. ***ii***, Spectrograms depicting theta and ripple frequency bands in the hippocampus. Levels of average band power are plotted in white, and periods of heightened theta (green) and ripple (pink) activities are highlighted via vertical shading. ***iii***, Raster plots of spiking in CA1 and PFC, with trajectories overlaid. Neural activity patterns along subspace dimensions are extracted from this spiking activity. ***D***, Communication subspace activity components from ***Ci*** are shown overlaid on the animal’s trajectory on a 2-D plot (gray zones are positions occupied on the track during a behavior session). The green and red circles denote start and end of trajectory. The scale indicates *z*-score of activity components, color-coded from blue (indicating low activity) to red (indicating high activity). ***E***, Communication subspace activity components (mean canonical variate activations) are shown averaged for all trajectories in a behavioral session, illustrating consistency with individual trial-based activations, leading to high and low activity zones on the track. Scale indicates *z*-scored activity. ***i***, ***ii***, and ***iii*** depict the spatial means for $UV_{1}$, $UV_{2}$, and $UV_{3}$ respectively. ***F***, Illustration of covariance structure and correlations of CCA shared-space components. The plot shows illustrative covariance of *U* and *V* components over one session of data for an animal. This matrix is separated by black solid lines into CA1 and PFC components, with the number of components equal the smallest rank of the two brain areas. Same brain area component correlations demonstrate orthogonalization of CCA components, whereas the CA1–PFC regions demonstrate the covariant overall relationship of a $U_{i}$ to a $V_{i}$ component. ***G***, Like ***F***, except zoomed in along the upper-right CA1–PFC quadrant of ***A***) ***H***, Correlation values between components across all animals, showing rapid drop-off with initial dimensions and exhibiting low-dimensional structure.

To sample the strongest time-varying activity in this communication space, we turned to a technique called CCA (Steinmetz et al., 2019; Veuthey et al., 2020; Kim et al., 2023). This technique, which exposes covarying activity patterns between two datasets, was used to identify major axes of interaction between the CA1 and PFC. This technique rotates the activity of two brain areas to expose their greatest covarying movements (Fig. 2*B*). Many top components contain correlated activity, but with far fewer components than neurons for CA1–PFC shared subspaces (Fig. 2*F*–*H*), forming a low-dimensional subspace. Here, we extracted the top two CCA canonical vectors to visualize their activity in correspondence with the raw LFP and spiking data. Each component has a hippocampal (*U*) portion and a prefrontal (*V*) portion that encodes how neurons that tend to covary in two regions activate over time, shown in Figure 2*C*–*E*. Figure 2*C* shows multiple outbound trials stitched together, with the magnitude of components shown in Figure 2*Ci*, with trajectory position overlaid as a gray line. Here *U*_1_ and *U*_2_ are the top two components for the CA1 portion and *V*_1_ and *V*_2_ for the PFC portion of the shared communication subspace. The observed activities of the components can be seen to be qualitatively repeatable across trials, with similar dynamics over the course of outbound trials from beginning to end. Figure 2*Cii* show theta and ripple frequency band spectrograms, and Figure 2*Ciii* shows aligned raster plots of spiking in CA1 and PFC ensembles. These top components exhibit consistent, repeatable deflections during SPW-Rs, during stillness periods in between ripples, and during running periods. Figure 2*D* shows examples of component magnitudes overlaid on an animal's trajectories, showing spatially localized activation during a trajectory. Figure 2*E* shows averaged components for all trajectories in a behavioral session, showing repeated high-activity spatial zones, illustrating that consistency of components across trajectories.

### Differential predictive performance and diversity of communication activity during rhythms

Next, we endeavored to understand and characterize the role of communication subspaces marked by rhythms. Given that we see a link between communication spaces and trajectory-specific position (Fig. 2), and several studies support links between task behavior and rhythms (Hyman et al., 2005; Haegens et al., 2014; Iwata et al., 2017; Moberly et al., 2018), we asked if there's any evidence that communication subspaces can be changed or modified by rhythmic activity. We primarily focused on theta and ripple oscillations given previous reports of relationships to memory-guided behavior (Jones and Wilson, 2005a; Benchenane et al., 2010; Sigurdsson et al., 2010; Jadhav et al., 2012; Joo and Frank, 2018; Hasz and Redish, 2020; Tang et al., 2021; Zhang et al., 2024). It is unclear how and if these states relate to the alignment of activity to the communication subspace and the implied shared activity mediated by these subspaces.

**Figure 3.**
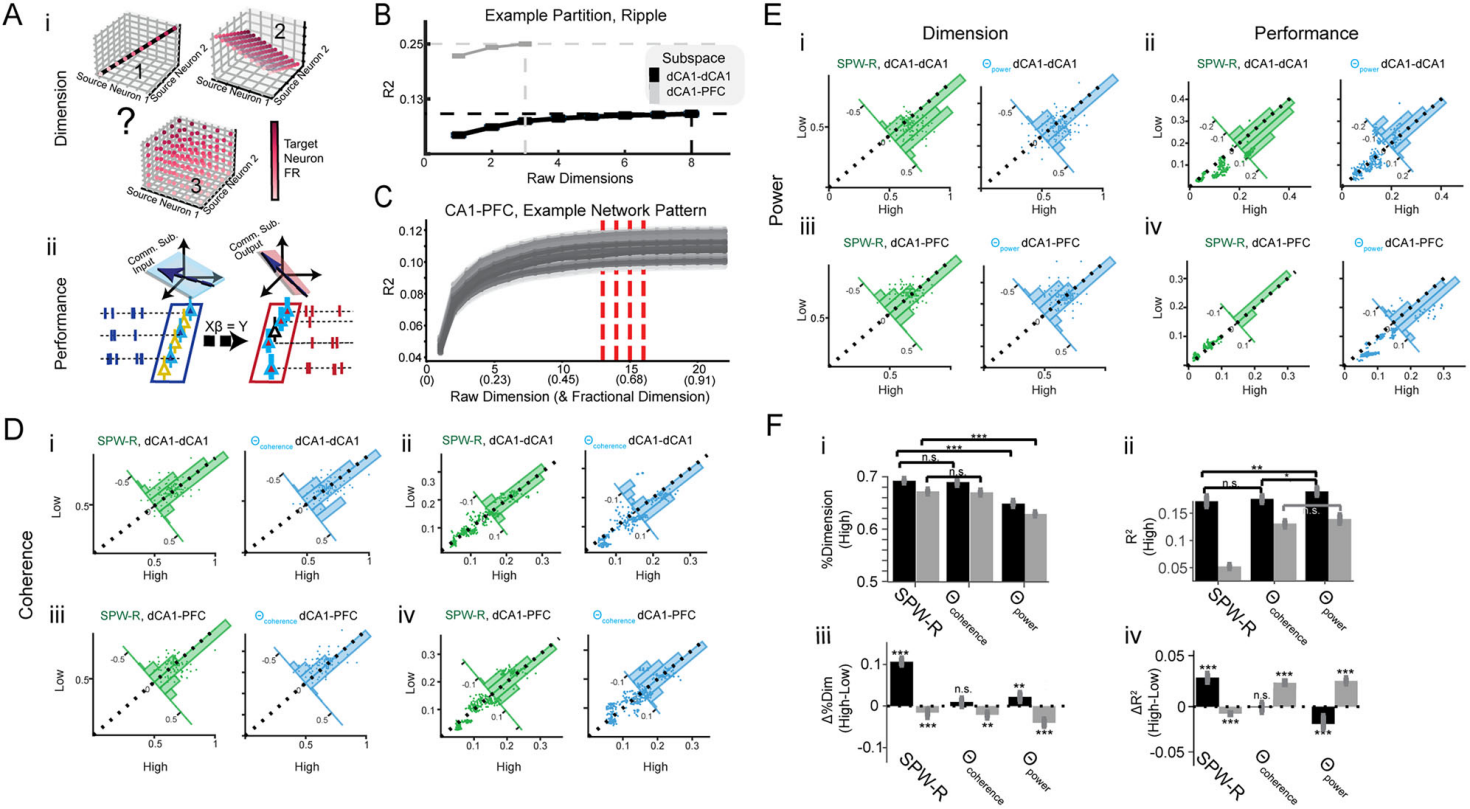
Assessing dimensionality and performance in rhythm-defined communication. ***A***, Schematic illustrating the two primary aspects being measured from network patterns within or between areas: the dimensionality and performance of rhythm-defined subspaces. ***i***, Dimensionality: the depicted subspaces represent possible communication dimensions within the neural data where target neuron(s) firing activity could potentially be influenced by various dimensionalities of source neurons. These three visualizations serve as exemplars, illustrating three potential dimensionalities to guide the reader’s understanding of the underlying principles and to preface what will be explored in sections ***B***–***D***. ***ii***, Performance: the graphic depicts a vector space defined by beta coefficients. The blue vector space symbolizes the input aspect, originating from the firing rate of source neurons (CA1). This input is processed through a linear model, encapsulated by the beta coefficient spaces, to project into the firing activity of target neurons. The output vector space is represented by target neurons, which can be located either in CA1 (the CA1–CA1 subspace) or PFC (the CA1–PFC subspace). ***B***, Measuring the communication dimension: this panel illustrates the process of assessing the communication dimension using a representative example. The *y*-axis depicts the model performance via coefficient of determination *R*^2^. A score of 1 represents the highest performance achievable with a full-rank model, while values deviating from 1 indicate fraction of variance explained. As dimensions (shown on the *x*-axis) are incrementally added, performance is evaluated until it aligns within one standard deviation of the full-rank model’s performance (dashed horizontal lines, asymptotic performance; dashed vertical lines, optimal dimension). The dashed lines are decided when performance does not differ from the full-rank model (within one SEM). In the depicted graph, intrahippocampal CA1–CA1 dimension is marked in black, while the hippocampal–prefrontal communication dimension is shown in gray. ***C***, For each animal and respective network pattern, data are generated as outlined in the text, with dimensionality resampled as in ***B*** for every partition. *X*-axis, the integer dimension represents the number of neurons in the partition, and the fraction represents the percentage of neurons used for asymptotic performance. Vertical red dashed lines indicate optimal dimensionality for 50 partition samples. ***D***, Dimensions (left column, ***i*** and ***iii***) and performance (right column, ***ii*** and ***iv***) of rhythm-defined communication for high versus low coherence during hippocampal SPW-Rs and theta. Each subplot depicts measurements for all 50 partitions per animal. Each subplot contains an overlaid inset histogram showing the difference for that partition measured during high and low activity pattern. A broad asymmetric lean of the histogram suggests a difference between high and low activity patterns. ***E***, Same as ***D*** for power instead of coherence. ***F***, ***i***, Comparison of dimension with network pattern type: CA1–CA1 dimension (black) and CA1–PFC dimension (gray). Error bars represent SEM. ***ii***, Model performance plotted against high network pattern activity: CA1–CA1 dimension (black) and communication dimension (gray). Error bars indicate SEM. ***iii***, Dimensional difference from high to low network activity pattern. ***iv***, Performance difference from high to low network activity pattern, with CA1–CA1 in black and CA1–PFC in gray. See Extended Data Figure 3-1.

To characterize whether the communication space is stable or modulated by these rhythms, we defined periods of high and low rhythmic network patterns for each frequency band, the power or coherence (Extended Data Fig. 3-1; Fig. 2*C*, periods). We then sought to measure two key aspects within our windows: the dimensionality, which indexes the diversity of orthogonal population vectors predicting the target, and, separately, the performance, measured by average cross-validated $R^{2},$ which indexes the subspace's ability to approximate information present in a source population about the target (schematized in Fig. 3*A*). Dimension was measured at the point where adding a dimension induced no significant performance increase. An example is shown for CA1–CA1 and CA1–PFC interactions in Figure 3*B*, exhibiting different dimensions for between-area compared with within-area dimension as in Semedo et al. (2019). Like in Semedo et al. (2019), for each animal we repartitioned source and target CA1 populations 50 times, both to control and match the number of target CA1 and target PFC cells, as well as to obtain a consensus of sample measurements that span all potential source CA1 neurons (Fig. 3*C*). These source–target partitions were measured for each collection of windows per network pattern event.

10.1523/ENEURO.0336-24.2025.f3-1 Figure 3-1 W**i**ndowing **of high and low network pattern events.** Download Figure 3-1, TIF file.

Figure 3*D*–*E* shows the values as histogram plots obtained for dimension (left columns) and performance (right columns) for coherence and power of rhythms (high vs low values), respectively, and Figure 3*F* shows the summary of the results. Our analysis of rhythmic communication patterns yields interesting insights, suggesting that not all rhythms express a given communication subspace equally. As previously seen, one tends to find different dimensions for within-region communication (Fig. 3*Fi*,*iii*, black, CA1–CA1) compared with across-region communication(gray, CA1–PFC) for each type of rhythm-defined window (Fig. 3*Fi*; two-sample *t* test; *p* < 0.001 for each; *N* = 350 partitions; 50 per animal; FDR corrected; *Q* = 0.001). This finding aligns with previous work (Semedo et al., 2019; Srinath et al., 2021) by showing reduced across-region communication dimensions for all rhythms compared with within-region dimensions. The higher dimensionality for CA1–CA1 is expected, since a large part of the CA1 network will contribute to within-region interactions compared with across-region CA1–PFC interactions. Notably, however, we make a novel observation that the presence of network patterns (high state minus low state) strengthens this difference in dimensionality (Fig. 3*Fiii*).

Beyond these similarities, network patterns also differ in their effects on the communication dimension. We observed a significant reduction in the diversity of both CA1–CA1 and CA1–PFC dimension diversity during high theta power states (two-sample *t* test; *p* < 0.001; *N* = 350 partitions; 50 per animal) relative to high SPW-R and high theta coherence states (Fig. 3*Fi*). Interestingly, the presence of theta coherence does not significantly modulate CA1–CA1 communication dimensions (*p* = 2.6710 × ^−1^), while the presence of SPW-R and theta power does. However, all three high states (e.g., high theta power) demonstrated reduced CA1–PFC dimension compared with low states, e.g., low theta power (Fig. 3*Fiii*), showing that the presence of a rhythm marked by high theta power leads to a lower-dimensional communication subspace, which can imply more specific interactions between neurons in the networks during the presence of the rhythm.

Apart from the diversity and dimension of network patterns, we can also investigate how these patterns influenced the predictability: how well is target region activity predicted from the source. Not surprisingly, CA1 cells better predict other CA1 cells than PFC cells (Fig. 3*Fii*) with significantly higher $R^{2}$ values during all three states (*p* < 0.001, all three rhythms). On the other hand, CA1–PFC rank regression prediction was maximal for theta-based patterns, and the presence of theta patterns (high/low comparison) increased CA1–PFC regression prediction (Fig. 3*Fiv*). In contrast, SPW-Rs strongly enhanced CA1–CA1 prediction. Overall, theta rhythms were associated with enhanced periods of predictable, lower-dimensional across–region communication. Meanwhile, SPW-Rs exhibited enhanced prediction more within CA1, with overall higher CA1–CA1 and CA1–PFC subspace diversity.

### Contributions of theta power and theta coherence to overall communication subspace over learning

We found it interesting that theta coherence exhibited no measurable performance gain in prediction when controlled against high theta power in this task (Fig. 3*F*). Previous studies either suggest CA1–PFC theta coherence to be periods of increased CA1–PFC spiking correlation (Benchenane et al., 2010) during choice periods, putatively for memory retrieval (Jones and Wilson, 2005b; Sigurdsson et al., 2010; O’Neill et al., 2013) or memory encoding (Battaglia et al., 2011). Some even suggest that theta coherence has no consequence on interareal spike correlations (Bygrave et al., 2019; Nardin et al., 2023). We therefore used the CCA method, which provides a temporal readout of shared/local communication without filtering window times. This method allows us to test whether theta coherence is temporally associated with communication along the top subspace components without the filtering window paradigm, which merely samples high and low states, and further, if so, does coupling between coherence and communication appear at a particular point within learning?

**Figure 4.**
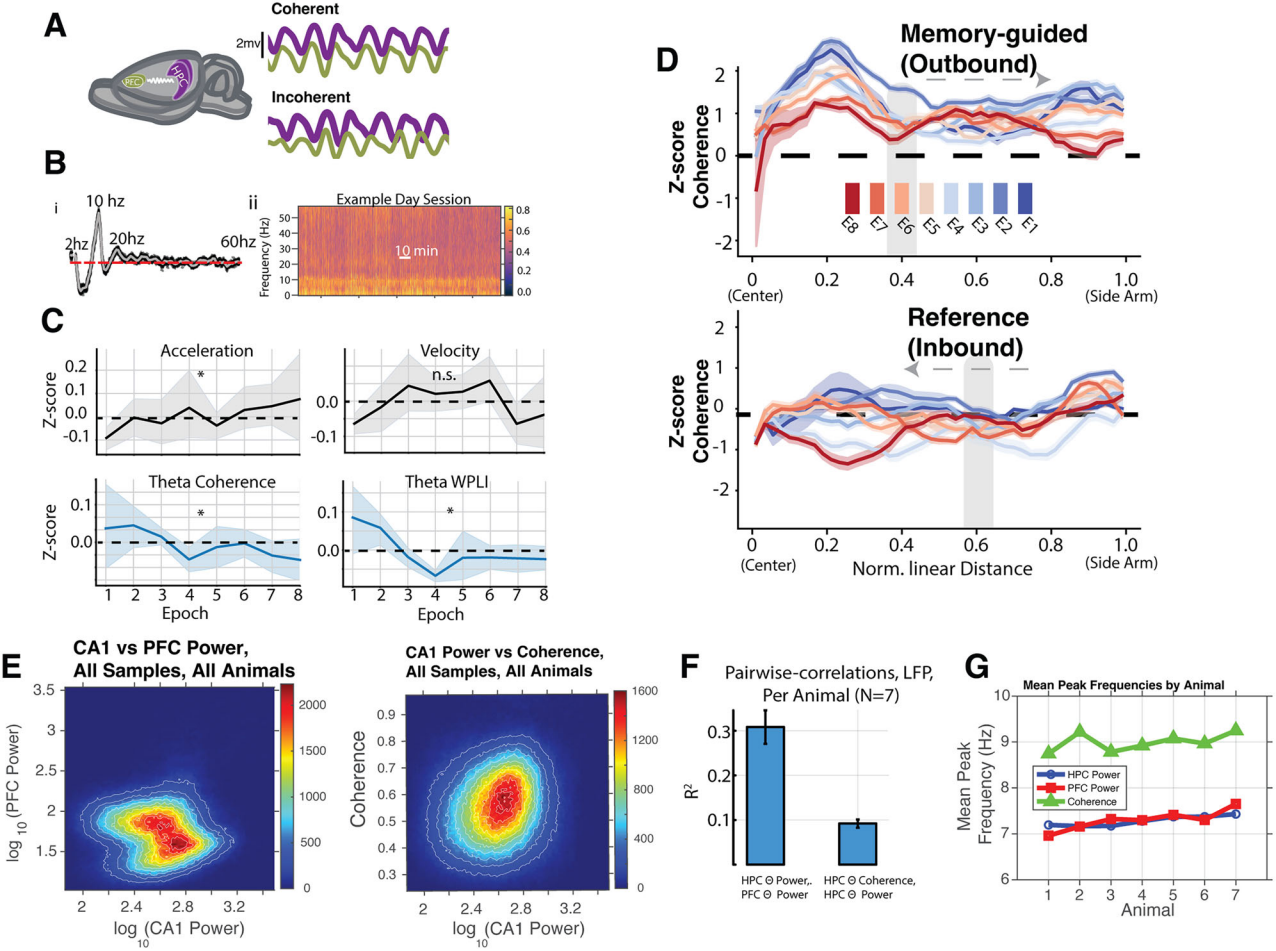
Dynamics of theta power and theta coherence over the course of learning. ***A***, The purple (CA1) and green (PFC) schematized traces are LFPs filtered in the theta band. Coherent theta activity holds a relatively constant phase lag relative to incoherent activity. ***B***, Panel ***i*** presents a mean coherence spectrum, highlighting prominent peaks in both delta and theta power ranges. Panel ***ii*** offers a snapshot of an entire session, showcasing the prevalence and interplay of strong theta and delta coherence throughout. ***C***, Panels depict epoch averages of theta coherence, theta WPLI, velocity, and acceleration, each normalized by *z*-scoring session values per animal. Each solid line represents mean values and shading the 95% confidence intervals for *N* = 7 animals. Notably, acceleration significantly increases (Mann–Kendall, *p* = 0.03; ANOVA, *p* = 0.64), while theta coherence significantly decreases for both multitaper (Mann–Kendall, *p* = 0.03; ANOVA, *p* = 0.33) and WPLI (Mann–Kendall, *p* = 0.03; ANOVA, *p* < 0.005) theta coherence. Velocity exhibits no significant changes (Mann–Kendall, *p* = 0.15; ANOVA, *p* = 0.09). ***D***, Comparative analysis of coherence against W-track linear distances, categorized into outbound (top panel) and inbound (lower panel) trajectories. The displayed curves, shaded for clarity using 95% confidence intervals, represent mean z-scored coherence values. A discernible decrease in coherence is evident across most track areas over time. ***E***, 2-D histograms showing distributions of CA1 and PFC theta power (left) and CA1 theta power with coherence (right). ***F***, Correlations between CA1 and PFC theta power are higher than correlations between CA1 theta power and theta coherence (Wilcoxon signed-rank test, *p* < 0.001). ***G***, Mean frequencies of peak CA1 theta power, PFC theta power, and CA1–PFC theta coherence. Coherence peaks are different from theta power peaks (Kruskal–Wallis test, *p* < 0.001).

We first characterized how theta coherence dynamics are modulated over learning in this task. To examine how coherence (Fig. 4*A*, schematic) changes over learning epochs, we utilized two methods of coherence measurement: multitaper coherence and WPLI (Mitra and Bokil, 2007; Vinck et al., 2012). Both showed strong low-frequency bands, most prominently the theta band (Fig. 4*B*). We found that overall coherence on the track dropped significantly over the learning period when averaged over all spatial positions rather than gaining steadily over time (Fig. 4*C*). We confirmed this general decline in coherence over learning epochs by *z*-score normalizing and min–max normalizing each animal's coherence and WPLI values (Fig. 4*C*).

Because different task phases or regions of the W-track may coincide with differing cognitive operations, e.g., coherence-mediated recall, we separated these measurements over space and trajectory type (Fig. 4*D*). Spatially binned coherence over epochs strongly confirmed a learning effect. Coherence began high in nearly all areas of the maze—most prominently in the center arm where animals presumably engage in working memory-guided decision-making. Moreover, coherence elevated throughout the entire outbound trial. However, over the course of the eight learning sessions, coherence declined. During outbound journeys, coherence remained above average levels, while during inbound returns, coherence was suppressed to around average. Overall, coherence declines from its peak well before animals hit peak performance at epoch 5–6 (Fig. 4*C*,*D*). This declining pattern of coherence over learning epochs can imply the role of coherence in online learning or encoding of the task rather than memory retrieval, aligning with prior theories (Battaglia et al., 2011). We also examined correlations between CA1–PFC theta power and CA1 theta power with CA1–PFC theta coherence (Fig. 4*E*–*G*). Theta power between CA1–PFC showed stronger correlations that CA1 theta power with coherence. We also observed that peak coherence between the regions was observed at slightly higher frequencies than peaks of power within CA1 or PFC (Fig. 4*G*).

**Figure 5.**
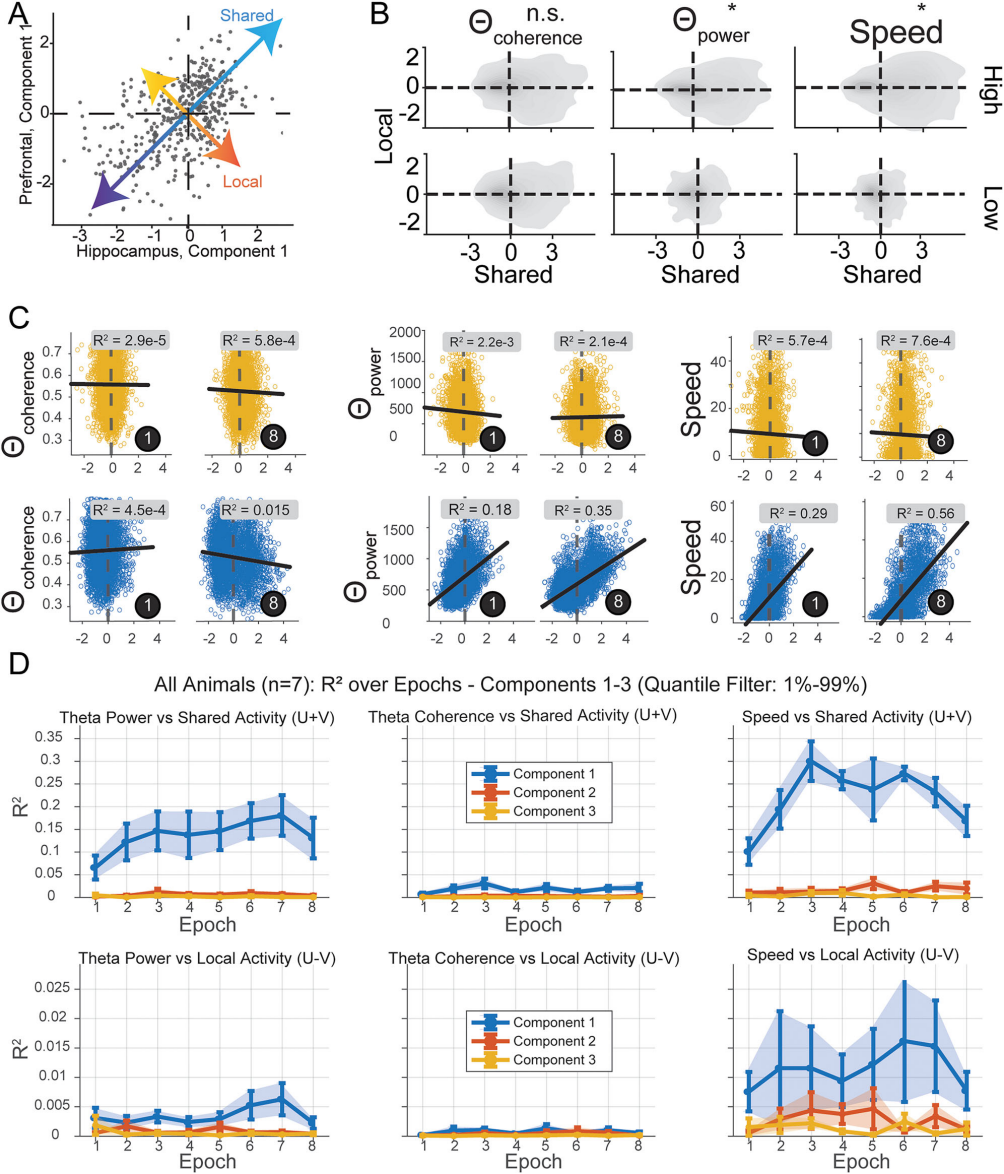
Change in shared activity correlations with theta power and coherence over the course of learning. ***A***, Example of shared versus local activity as in Figure 3. ***B***, Kernel density plots showing the distribution of samples for windows of high versus low activity for theta power, theta coherence, and speed. The densities indicate that the range of observed values in shared and local activity depends more strongly on power than coherence. * indicates *p* < 0.001 via the permutation test of the univariate shared axis distribution. ***C***, Scatterplots of shared/local components for an example animal from the first CCA component (*x*-axis) versus theta power/coherence/movement speed (*y*-axis) levels. Samples are taken for all times. Coefficient of determination is displayed atop each subplot. The top row depicts local components (yellow), and the bottom depicts shared components (blue). ***D***, Summary of the coefficient of determination values found for (*N* = 7) all animals for the top three CCA components for shared (top row) and local (bottom row) subspaces, over the course of learning. The shaded region depicts 95% confidence interval. For the top CCA component, the CCA relationship with theta power and speed is much higher than theta coherence. The other components (Components 2 and 3) contribute much lower values than the first component.

We next turned to characterize the fluctuations of the top CCA components in shared subspaces and local activity (Fig. 5*A*) with respect to CA1 theta power and coherence. Significantly, the range of canonical variate values reflecting shared activity substantially varied between high and low theta power states, which did not occur for unreciprocated, local activity; however, these same values of shared activity did not vary as much with different theta coherence states (Fig. 5*B*). Since this relationship may change over epochs, in a more refined manner, we trained a linear model once per epoch using the top shared communication components to predict theta coherence. Examples for an animal in early behavioral Epoch 1 and late behavioral Epoch 8 is shown in Figure 5*C* (local activity in yellow showing low correlation, shared subspace activity in blue showing high correlation for theta power and speed). Data for all animals are shown in Figure 5*D*, with shared-space Components 1–3 in the top row and local Components 1–3 in the bottom row. Over all the animals, theta coherence in shared space exhibited a low relation with aligned communication ( R=0.03 for shared component 1; Fig. 5*D*, middle). In contrast, all animals exhibited a strong relationship with theta power ( R=0.25 for shared component 1; Fig. 5*D*, left), with several individual animals showing very strong relationships. This theta power relationship grows over learning epochs, asymptoting around the time (Epochs 3–4) that place fields and population vector codes stabilize (Tang et al., 2021; Zielinski et al., 2021). Notably, the animals exhibit no trends in mean velocity (Fig. 4*C*; ANOVA; *p* = 0.14; Mann–Kendall; *p* = 0.90). Acceleration, on the other hand, exhibits a minor trend (Fig. 4*C*; Mann–Kendall; τ = 0.64; *p* = 0.03) over learning across animals, though we did not find individual epoch acceleration to be well differentiated across epochs (Fig. 4*C*; ANOVA; *p* = 0.65).

### Shared communication subspaces but not local subspaces encode task-relevant behavior

**Figure 6.**
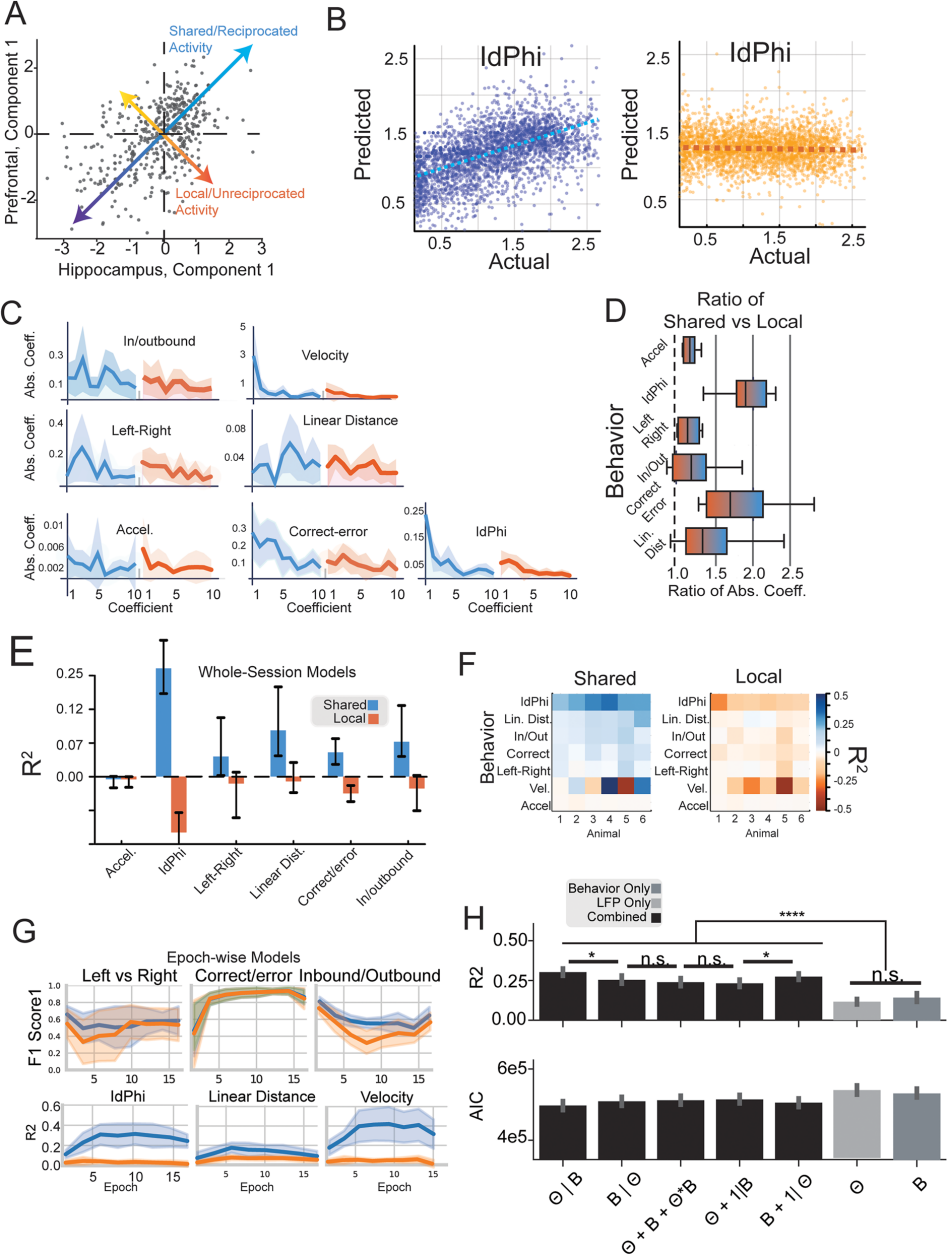
Shared CA1–PFC activity predicts task behavior better than local activity. ***A***, A scatter of time samples on the *U* (CA1) and *V* (PFC) axes. The blue arrow illustrates aligned activity between CA1 and PFC, while the orange arrow indicates unreciprocated CA1 activity with corresponding PFC movements. ***B***, Outcomes from the Bayesian linear model predicting IdPhi, a metric indicating VTE. Analysis contrasts reciprocated (blue) versus unreciprocated (orange) movements. ***C***, Display of linear model coefficients corresponding to seven distinct behaviors on the W-track task. Coefficients are color-coded: shared (blue) and local (orange). Coefficients, resampled via Markov chains, come with shaded 95% confidence intervals. ***D***, A depiction of the ratio between shared and local cumulative coefficient magnitudes. A unity reference (1) is marked by a dashed black line, representing equal contributions. ***E***, $R^{2}$ values (*y* values) for each linear model predicting a behavior (*x*-axis) from canonical variates. Shared activities significantly predict task-relevant behaviors above zero, while local activities do not. ***F***, A breakdown of mean $R^{2}$ values as in ***E*** for individual animals, categorized by aligned (left panel) and orthogonal (right panel) components. ***G***, Performance of models trained over individual single-day learning epochs. The bottom panel shows $R^{2}$ values for continuous variables over epochs, while the top panels display *F*_1_ scores for classification variables. Shared components (blue) tend to outperform local components (orange) in predicting task-relevant behaviors, except correct/error. Shaded regions represent 95% confidence intervals. ***H***, Comparison of models predicting the top CCA shared component from various combinations of theta power (θ) and continuous behavior (B) information. Models are specified using *R*-like formula notation, where hierarchical terms are indicated by the vertical bar operator (|) and interaction terms by the asterisk operator (*). For each model, the top value represents the $R^{2}$, and the bottom value represents the Akaike information criterion (AIC), which accounts for model complexity and goodness of fit. Models incorporating both behavior and theta power information consistently exhibit better performance in terms of higher goodness of fit $R^{2}$ and lower AIC values compared with models using only one source of information. Error bars depict 95% confidence intervals.

Finally, we sought to better understand whether these fluctuations encode task-relevant behavior. To that aim, we adopted and trained a simple linear model, resampled, and retrained repeatedly with MCMC techniques. We used two activity categories related to the top 10 canonical CA1–PFC variates in the communication space: shared and local activity. More conceptually, aligned activity refers to source and target neural movements that are reciprocated and coincide with one another. In contrast, local activity describes a situation where either the source or target has varied in the communication space without commensurate activity in the other area. Like the activity in Veuthey et al. (2020), similar to private activity, it may be indicative of future communication but is not yet a shared fluctuation. Figure 6*A* displays the activity of CA1 and PFC projected onto the top canonical variate. The U (*x*-axis) encodes how much CA1 activity matches the communication covariate vector, and the V (*y*-axis) encodes how much PFC matches the same covariate. If we look at the projection onto the unity line, this is where changes (or motions) of neural activity in one brain area reciprocated into the other brain area, i.e., shared activity. The orthogonal axis to the shared motion shows unreciprocated motions, i.e., local subspace, positive or negative, imply population vector motions along one area's communication axis without concomitant expected motions in the other brain areas.

We sought to understand if activity along several possible shared and local activity axes explains behavior. Thus, we desired to train a linear decoder to predict behavior from the top K shared or local activity components. CCA exhibits as many canonical covariates as the smallest neuron count (the minimal rank of the brain areas), but in practice, far fewer contain significant shared activity. Most animals express <10 significant axes, and therefore, we used the top 10 covariates aligned/reciprocal activity and orthogonal/nonreciprocal activity to train a linear decoder to predict task behavior. Many task-relevant behaviors could be approximated with this very crude model (*N* = 6 animals) but only using the reciprocated communication activity (Fig. 6*B*,*E*,*F*). Task-relevant variables include the phase of the task (outbound/inbound), linear distance along the track, IdPhi (integrated local head direction changes), the turn direction, and the trial's correct versus error state. Key among these, IdPhi (Fig. 6*B*) measures an animal's integrated head deflections locally in time and has previously been used to index vicarious trial and error (VTE) and deliberative decision behavior (Papale et al., 2012; Schmidt et al., 2013; Redish, 2016; Santos-Pata and Verschure, 2018); this index of VTE was highly represented in shared CA1–PFC activity across animals. Such predictive patterns are replicated across animals (Fig. 6*F*).

In addition to this task-relevant aligned encoding, we also examined the unreciprocated, local communication (Fig. 6, orange). In every case, surprisingly, the local subspace's unreciprocated activity provided little to no predictive power. This can be seen in the flat response of predicted IdPhi versus actual (Fig. 6*B*) as well as the poor determinacy over task variables in Figure 6, *E* and *F*. Linear models trained to predict behavior with local activity produced muted beta coefficients for local components (Fig. 6*C*) relative to reciprocated components (Fig. 6*D*). These findings collectively imply that while shared dynamics between CA1 and PFC significantly predict behavior, the individual subspace activity within the regions does not, highlighting the unique behavioral significance of communication space-driven shared activity.

To examine whether learning changed this behavioral prediction, we turned to training GLM models in an epoch-wise fashion (Fig. 6*G*). We created a series of cross-validated models within each epoch trained on the top 10 CCA components predicting six of the behaviors. Continuous behaviors, such as IdPhi, linear distance, and velocity, exhibited enhanced predictive performance $R^{2}$ in the shared versus local components, which rose over the learning period and stabilized in the well-learned period. For the classification variables, local and shared produced more similar predictions across epochs as measured by *F* scores. Only the correct–error classifier increased over epochs, and only the in-/outbound classifier produced a period of significant difference.

Given communication strongly indicated behavior (Fig. 6) and our previous analysis showed increasing correlation of the top CCA component with theta (Fig. 5), we asked whether the LFP effects could be entirely explained by behavior, vice versa, or required both. Perhaps behavioral state determined LFPs, and the former differences (Figs. 3, 4) could be explained solely by behavior. To this end, we created a series of models to explore the effect of behavior alone, theta alone, or models that combined information from both sources predicting the contiguous behaviors discussed above (speed, IdPhi, and linear distance). Notably, behavior and theta power generated similar performance for the top CCA component when pooling the prediction of velocity, linear distance, and IdPhi prediction (Fig. 6H), but to our surprise, all models that combined both sources of information created better predictions with lower AIC (*t* test2; *p* < 0.01 for all interaction versus univariate; Fig. 6*H*). The improved predictive power from interactions suggests that behavior and rhythms, while correlated, have independent influences on communication subspace activity.

**Figure 7.**
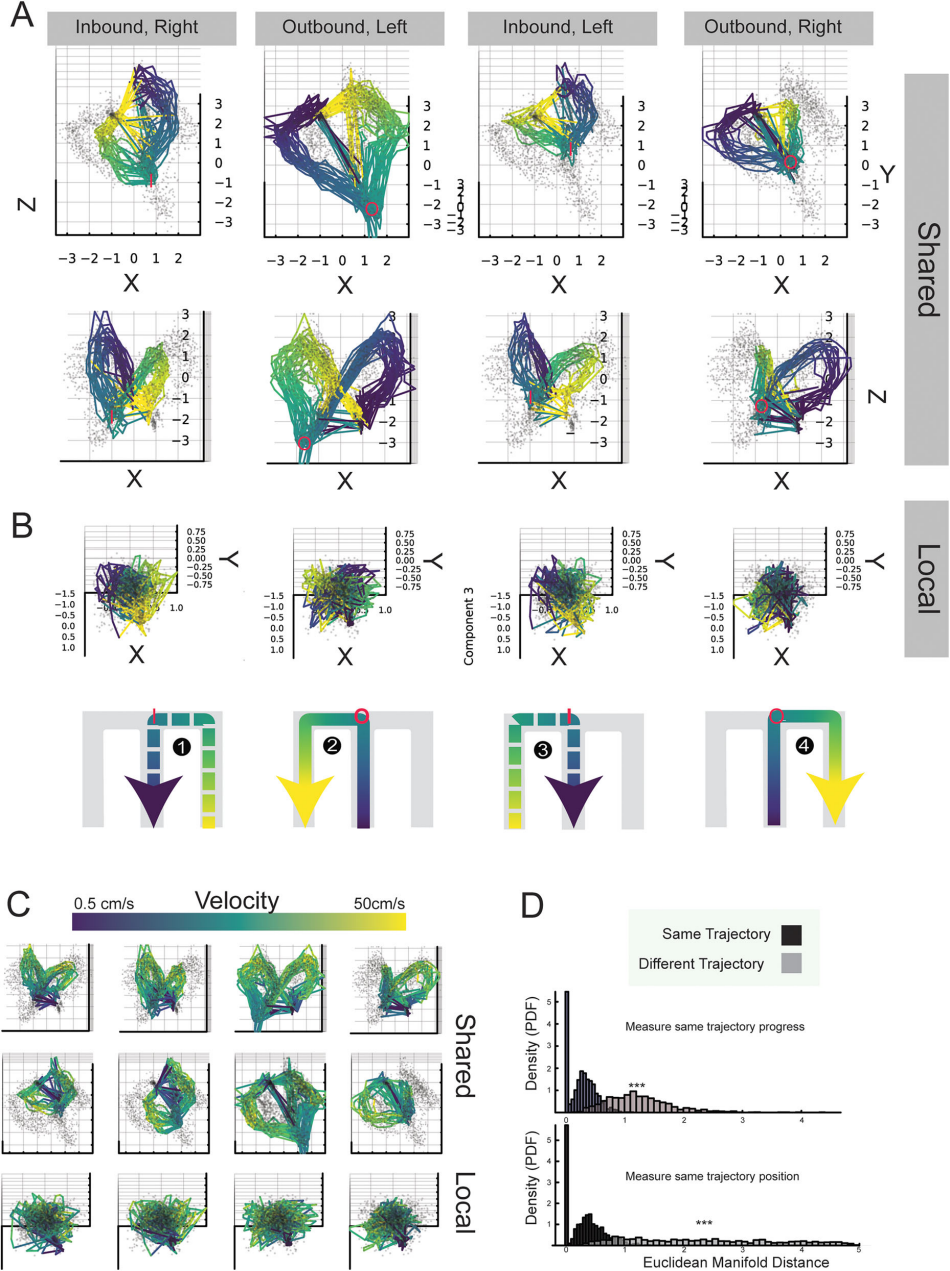
The communication subspace organizes trajectories in nested ring structures. ***A***, Combined animal communication shared subspace. Two different views of the three-dimensional subspace are shown in the two rows. Trajectories of each type were ordered, binned, and concatenated across animals to help visualize whether the communication subspace organizes activity into manifold and whether that manifold organizes behavior. In this case, we color-labeled the manifold by the linear distance bins (center, violet; side arm, yellow), positions shown in the panels below (***B***) for local activity. The shared communication activity organizes trajectories into rings, with remote-like bisection events between choice and reward locations. Each trajectory is colored in accord with panels below (***B***), while other trajectory points are depicted by small gray scatter points. The choice zone is demarcated by a red “o” symbol for outbound and “I” for inbound. This choice locus differs clearly across the many samples of each trajectory type and, together with other spatial differences, explains the ability of linear decoders to sense behavior in this space. ***B***, This structure is mostly absent for local communication activity, activity matching the communication subspace but not coordinated across the two areas. A small amount of positional clustering can be observed. ***C***, This shows the same manifolds as in ***A*** and ***B*** but colored by the average animal velocity. Ring structure bisection events can be seen as lower velocity states. ***D***, Probability density normalized histograms of manifold Euclidean distance within (black) and between (black) communication manifolds, where distances are taken for samples sharing a linear distance. See Extended Data Figure 7-1 and Movies 1 and 2.

Lastly, given that communication subspace activity well explained behavior, we wondered whether the subspace manifold structure itself appeared to structure behavior into an orderly manifold. To this aim, we created a pooled dataset across animals based on the trajectory type in the manner of Tang et al. (2023), and then we color-coded the CA1–PFC shared and CA1–PFC local activity manifold by the associated mean behavioral labels (Fig. 7*A*,*B*; Movie 1). This revealed the presence of differential cyclic structures for shared activity manifolds—structures not apparent for local activity manifolds. Each shared activity manifold possesses a unique cyclical path proceeding clockwise through communication space (looking toward the midline crease). Nested ring sizes signaled the trajectory type (inbound/outbound) and left versus right. This was confirmed by measuring the difference in Euclidean distance between points sharing linear distance within and between communication manifolds (Wilcoxon rank sum *p* < 0.001, between vs within distance 95% CI 1.90–2.06; Fig. 7*D*). Taken together with the clockwise observation, points on different rings are neighboring not by linear distance from the center but rather along trajectory progress, with rings folded at the choice and reward points. Furthermore, robust separation occurs at the choice point between manifolds.

10.1523/ENEURO.0336-24.2025.f7-1 Figure 7-1 **Attractor Hypothesis.** The four trajectories are schematized. Inbound is shown in dashed line style, outbound in solid line style, leftward is red, and rightward is blue. The trajectories form neighboring rings—all proceeding clockwise **(**Figure 7**)**. Different trajectories are labeled 1-4 near their respective choice point schematics. Active start/end and choice points are shown in white. Though we have not explored the initial positions, we hypothesize that neighboring ring attractors could distinguish trajectory class and may terminate near the start of the upcoming trajectory. In this manner, such CA1-PFC coactivity could implement a form of task logic in the communication space. Download Figure 7-1, TIF file.

**Movie 1.** CA1–PFC shared activity manifolds color-coded by trajectory types. Three-dimensional visualization of the top three CCA components from pooled data across all animals (*N* = 6). The top panel row shows CA1–PFC shared communication subspace, and columns represent trajectories (left-to-center, center-to-left, right-to-center, center-to-right). Samples are color-coded by linearized spatial bins along the linear track (violet, terminal center arm; yellow, terminal side arm). The shared subspace CCA geometry exhibits distinct cyclic ring structures that map systematically to behavioral trajectories. In contrast, local CA1 activity shows dispersed organization without clear ring geometry, as seen in Figure 7*B*. [View online]

**Movie 2.** CA1–PFC shared activity subspaces color-coded by velocity. Three-dimensional visualization of the top three CCA components showing the CA1–PFC shared communication subspace with activity color-coded by running velocity (violet <5 cm/s to yellow >30 cm/s). Low-velocity, pinched ring regions (violet) appear consistently at reward locations and choice points where animals pause or slow. High-velocity, curved-up regions (yellow) correspond to active running along maze arms and stem. The systematic velocity gradient along the ring structure indicates that CA1–PFC communication CCA geometry corresponds well to locomotor state, a feature that is absent in local activity spaces, as seen in Figure 7*C*. [View online]

These rings interestingly exhibit bisection. The activity at the outer wells (center and side arm) jumps to the other side of the ring closer to the choice point. From a neural geometry perspective, these points (reward wells and choice points) are closer to each other on the third axis, where the rings are folded downward, increasing their similarity and signaling, potentially reducing the energy landscape barrier for neurons in the communication space moving between these points. We also examined this space labeled by average velocity. Notably, the activity bisecting between the reward arm and choice regions is associated with regions of lower velocity (Fig. 7*C*; Movie 2). In summary, labeling the shared activity manifold by speed and space makes it apparent “why” these spaces may produce behavioral prediction, even with simple linear decoders.

## Discussion

Our examination of hippocampal subspace dynamics during memory-guided behavior yields several key insights. We found that shared CA1–PFC activity within low-dimensional communication subspaces predicts task behavior (Figs. 5, 6). Our results suggest that this is because CA1–PFC shared activity forms organized ring-like manifolds arranging various behavioral states (trajectories) in the W-track task. Furthermore, dimensionality reduction techniques revealed that prominent rhythms modulate subspace properties (Fig. 3). Notably, theta periods exhibited increased predictability of CA1–PFC activity and reduced CA1–PFC communication dimensionality, compared with greater intra-CA1 diversity during SPW-R events (Fig. 3). Surprisingly, we found that theta coherence did not coordinate shared subspace activity as well as theta power in this task (Figs. 4, 5).

Memory-guided behavior requires hippocampal–prefrontal interactions (Floresco et al., 1997; Shin and Jadhav, 2016; Maharjan et al., 2018; Rezayat et al., 2022). Our approach sought to measure activity across networks via shared communication subspace versus local activity via private communication subspaces (Figs. 2, 5–7). This approach directly suggests that shared information is better related to immediate behavior compared with locally held information. This does not preclude such local activity from having delayed effects on memory-guided behavior; indeed, Veuthey et al. (2020) found that local, unreciprocated activity often precedes changes in shared activity by up to a second and frequently follows changes during early learning in the motor cortex. In a similar vein, we saw that the local manifolds appear rather disorganized, though future studies may find greater order when examining local spaces against time-lagged behavior; these spaces may contain prospective or retrospective information yet to be coordinated across areas. An example of this time-lagged interaction occurs in Kaufman et al. (2014), Elsayed et al. (2016), and Veuthey et al. (2020). We have shown here, on the other hand, that shared space well organizes the animal's task, track, and velocity space. These structures contain potential clues for how CA1 and PFC networks coordinate to implement the W-track task rule. The ring-like manifolds emerging out of the shared activity differ by task phase (Fig. 7) but potentially relate to geometric structures seen in these areas individually (Tang et al., 2023). Notably, the neural activity in the communication space indexes the current location and trajectory subtype (Extended Data Fig. 7-1). Neighboring trajectory loops could act like attractors—paths that neural activity may bias to remain near based on the shared coactivity. Then progress coding may bear some resemblance to that noted in PFC by El-Gaby et al. (2023), who found cells indexing trajectory progress in PFC. However, in our study, such information emerges in CA1–PFC coordination.

Differences also emerged at the intrahippocampal level between theta and ripple states. Intrahippocampally, our results revealed differences in diversity (Fig. 3), with ripples exhibiting a larger increase in diversity. One potential explanation is that distinct neuron populations activate within these rhythms, such as CCK+ and OL-M cells during theta power versus PV+ cells during SPW-Rs (Klausberger et al., 2003; Somogyi et al., 2014). Differential neuronal inhibition levels likely contribute to the differences in spreading intrahippocampal activity observed during ripples and theta states. Much like in the case of the visual cortex (Semedo et al., 2019), we note that the dimensionality changes in more subtle than drastic ways. The overall changes tend to be on the scale of 10% differences in assembly composition. Likewise, the shared spaces have similar magnitudes of predictability, from 5 to 20%.

Prior studies posit that theta coherence aids in retrieval processes in alternation tasks. However, these results on the W-track alternation task do not appear to support that view. Prior results with these data also failed to find a difference in theta coherence between correct and error trials in this task (Tang et al., 2021). These results resemble older theories that propose theta coherence is conducive for plasticity and synaptic weight changes during early learning and the theta rhythm parcellates ideal windows for inducing LTP and LTD (Hyman et al., 2003; Dragoi and Buzsáki, 2006; Buzsáki and Moser, 2013; Wilson et al., 2015). This can allow theta coherence with another region to take advantage of this window for linking assemblies of ongoing experiences. Thereafter, ripple-associated reactivation can stabilize weakly linked traces (Benchenane et al., 2011). It is possible that these results are specific to this experimental design, with rapid single-day learning in the W-maze alternation task.

Our study has some key limitations. The analyses were restricted to only awake brain states. Possibly, important population vectors and local/shared interaction balances substantially differ between sleep and wake. Examining communication architectures during sleep presents an important future direction for fully characterizing hippocampal subspace dynamics. Additionally, prior work has found limited impact of nonlinearities on the dimensionality and geometry of visual cortical communication subspaces. However, using residual rather than total activity helps remove nonlinearity. The W-track task, however, lacks the regular trial structure permitting residual removal previously used. So, we focused on full *z*-scored spiking, which subtracts a mean. Total activity previously yielded a similar result (Semedo et al., 2019). Still, nonlinearities can alter linear approximation in nuanced ways.

Lastly, we characterized the effects of rhythms, behavior, and changing spike alignments on a stable, session-wide subspace, which has pros and cons. The pro observes how activity aligns with an overall fixed state of CA1–PFC coordination. The con is an inability to examine acute but stable coordination structures in different epochs, especially early epochs. Future studies could utilize a temporally windowed approach to understand how temporary communication dimensions arise and either stably incorporate them into the already stable CA1–PFC structure or fail to do so. Separate epoch analysis may reveal learning-related changes in the rhythmic modulation and behavioral structure of these shared CA1–PFC spaces. It is also possible that the differences in communication subspaces observed for theta and ripples are due to state differences for these two rhythms which occur during immobility versus moving and reflect online versus offline dynamics. The lower number of PFC neurons may also constrain the number of subspace dimensions observed.

One difficulty is that our recordings contained limited numbers of certain events like ripples, presenting analysis challenges. Using lower quantile thresholds to split by epoch could help overcome limited events like ripples for analysis. This task also involved two dissociable rules with likely differential working and reference memory demands. Comparing subspace modulation by the rule type presents another interesting direction, including testing the specificity of theta's growing communication influence. More broadly, future work could manipulate rhythms to causally probe their impacts on dimensionality. Physiological identification of neuron types in the subspaces could reveal a cause for warping or angular changes. Much remains unknown regarding how subspace topography sculpts routing, presenting exciting frontiers for mapping this structure–function landscape.

These results provide evidence for the importance of reciprocal interaction on behavior and the influence of rhythm on communication subspaces, particularly theta power, not coherence. Together, these observations advance an understanding of how oscillatory states, memory-guided behavior, and spiking-based communication motifs entangle during the routing of information through neural populations.

## Synthesis

Reviewing Editor: Mark Laubach, American University Decisions are customarily a result of the Reviewing Editor and the peer reviewers coming together and discussing their recommendations until a consensus is reached. When revisions are invited, a fact-based synthesis statement explaining their decision and outlining what is needed to prepare a revision will be listed below. The following reviewer(s) agreed to reveal their identity: James Hyman, Antonio Fernandez-Ruiz.

Thank you for sending your study to eNeuro. Your paper was reviewed by two experts and their reviews are given in full below. Please revise your manuscript accordingly and it will be sent out for a second round of review.

Reviewer 1

This paper presents data from recordings of mPFC and CA1 during performance of a W maze task. The author's main analyses rely upon cell assembly detection through CCA-based methods. They then examine how other factors connect to assembly activation and information coding. They found that shared space activity between mPFC and CA1 better predicted behavior than local activity. Shared space communication was modulated by SWR, CA1 theta power and running speed, and not as much theta coherence. In fact, they found coherence decreased over learning while power increased. They then show that the shared space forms ring-like structures corresponding to task progression, that was not present in the local space. Overall, this is an excellent paper that is well analyzed and presents important, novel findings. I had some difficulty making my way through the paper due to the density of the figures, the lack of axis labels, the over use of supplemental figures, and the lack of clear narrative when discussing the layered analyses utilized. With revisions that will make the paper more accessible for the reader, this will make a fine addition to the literature.

Main Points:

One of the articles main claims is that theta coherence is not associated with better memory performance (Fig. 4). They support this claim based upon the lack of coherence increases over learning (good) and the lack of correlation between the top CCA component and coherence, while theta power and speed were correlated with this component. Given the well established relationship between CA1 theta power and speed, and the dominance of theta within CA1 LFPs, it is not that surprising that the top component would reflect this. Coherence relies upon PFC theta as well, which has a much more tenuous link with running speed, and is less dominant in the PFC LFP. It seems reasonable that coherence would be more likely to correlate with other lower CCA components. How does the relationship look for components 2-10? Since early in learning speed is lower and also theta power is lower, but theta coherence is higher, is this just because less speed allows coherence to influence the top component. I guess, overall, what I'm worried about is whether the hippocampal signal is so monopolized by theta power, it's masking any contributions in the shared space of theta coherence. Also, given that the authors explored more than just CCA component 1 in other analyses, why only look at # 1 here? I am also curious what effect PFC theta power when examined with this analysis. If a similar pattern holds, then it would support the author's assertion that power is the key element for sustained performance. Lastly, the authors were very thorough in all their other analysis, but seem to have been very quick with this analysis, which is potentially the controversial claim in this paper. I think when making such claims it is important to go above and beyond to show that it is correct. The coherence results alone are impressive, but the thrust of the claim comes down to the shared communication space. Showing there's nothing on any of the components would make the author's conclusions stronger.

Explanation of subspace analysis needs to be clearer in the text. The explanation in the figure legend is better, but a more direct walk through of these concepts is needed, since this is the basis of all the rest of the analysis.

The analysis of dim reduction in Fig. 3E is very difficult to get through. I'm a bit lost on how there are both theta and SWR results and also dim removed. Where were dimensions for SWR and theta identified so they could be removed.

Minor points

L 264 - typo

L343 - typo

L359 - this is an interesting finding, but it is very hard to decipher. A sentence taking this finding back to the data (in terms of inter/intra areal communication) would really help the readers understand this point better.

L397 - typo

L513 "Figure A"?

L620 typo

What are the two rows of Fig. 6A - are these examples from two different example assemblies? This should be explained in the figure legend.

L699 seems like a claim that is a bit too far for the results presented here.

Reviewer 2

This paper investigated the coordination of population activity across prefrontal cortex (PFC) and hippocampal CA1 areas durign spatial learning in rats. Specifically, the authors ask the question of spiking population dynamics define communication subspaces across these structures and how these are related to hippocampal network rhythms such as theta oscillations and sharp-wave ripples (SWRs). Communication subspaces have been analyzed to characterize functional interactions across brain areas durign perceptual tasks and other cognitive functions, but they have not yet been explored in depth in relation to learning and oscillatory interactions. Therefore, the approach of the authors is original and fills a gap in the field. Taking one step further from the established methods for low-dimensional communication subspaces, this work separated and characterized the reciprocated from the unreciprocated local activity in the low-dimensional communication subspace. Importantly, the results highlights that the low-dimensional communication subspace codes for spatial and task-relevant information, specifically by the shared activity instead of the local activity in the communication subspace. The findings also suggest that behavior and rhythmic states both contributed to shaping the communication subspaces. To reach these conclusions, the authors analyzed a previously published and very high-quality dataset of neural ensemble recordings in behaving rats. The methods used are sound and the results presented support the authors interpretation. The results presented here would be an important contribution to the field.

There are some issues that need more clarification before publication.

• The definition of local activity in the communication subspace needs to be clarified. This paper made a distinction between the private subspace and local activity in the communication subspace. This definition of local activity is different from previous work in the field, including some of the references (Kaufman 2014, Veuthey 2020), where local activity usually refers to activity in the private subspace (the null space of the communication subspace).

• The dimension of the communication subspace was determined with reduced rank analysis, and one can appreciate the dimension changes with different states. How about the subspace found by CCA? CCA finds the orthogonal basis for the correlation matrices; it might thus contain the information from the private subspace if not well controlled (Veuthey 2020). Please provide some validation for this.

• Theta coherence and hippocampal theta power are related. It would be useful to see a joint distribution of the two. In addition, does the theta coherence and power peak at the same frequency?

• The reduced rank regression in line 343 and 354 both uses ordinary least squares estimator, instead of a ridge regression, which uses Tikhonov regularization. The authors implied that communication happens in a sparse, low-dimensional communication subspace. However, a low-dimensional manifold does not necessarily imply sparsity. Neither of the methods implemented here (RRR or CCA) assumes sparsity. Can the authors clarify this point?

• Some of the figures and analysis are a bit dense and will benefit from more detailed explanations and interpretation. An example of this is Figure 2. First some panels are missing scale bars (D) or those bars are missing corresponding label (E). The description in lines 236-238 is not clear enough and would need more extensive explanations.

• Some of the results would benefit from a clearer interpretation. For example, is not very clear what was learnt from the analysis depicted in Fig. 3E-H.
