# Supplementary material for: Hippocampal–Prefrontal Communication Subspaces Align with Behavioral and Network Patterns in a Spatial Memory Task
Source: eNeuro. 2025 Sep 18;12(9):ENEURO.0336-24.2025. doi: 10.1523/ENEURO.0336-24.2025 (PMC12463551; doi:10.1523/ENEURO.0336-24.2025)
Supplement: Extended Data 1 — Download Extended Data 1, ZIP file. [file eneuro-12-ENEURO.0336-24.2025-s006.zip › BNPCS/Notebooks/SemedoPaperFigures_extant.pdf]

```
% paths
if ispc
    path = ("C:\Users\BrainMaker\commsubspace\hash_multiEpoch");
elseif ismac
    path = "~/Data/commsubspace/hash";
end
addpath(path)

addpath(genpath('utils')); % all folders in utils added, including semedo
code
```

```
% load
multi_epoch = true;
if multi_epoch

    load("Tablerow_multiEpoch.mat");
else
    load("Tablerow_3_FRfixed.mat");
end
% TABLE : ACQUIRE RUNS
% ----------------------
% Determine keys to use : you can use this string to arbitrarily select rows
%   each item of the filtstring is a property to select. $x pulls the x
%   column and applies the test shown
filtstring = ["ismember($animal,
[""JS21"",""ZT2"",""ER1"",""JS14"",""JS13"",""JS17""])",...
    "$spikeBinSize==0.15",...
    "$numPartition==50",...
    "$quantileToMakeWindows == 0.85",...
    "all(cat(1,$winSize{:})==[-0.15,0.15],2)"];
% Get the proper keys
if ~multi_epoch
    matching_runs = query.getHashed_stringFilt(Tablerow_3_FRfixed,
filtstring)
else
    matching_runs = query.getHashed_stringFilt(Tablerow_multiEpoch,
filtstring)
end
```

```
Filtering with:
T(ismember(T.animal, ["JS21","ZT2","ER1","JS14","JS13","JS17"]) & T.spikeBinSize==0.15 & T.numPartition==5
matching_runs = 12×20 table
                                                                                                          ...
```

	animal	generateH	epochsSelected		samplingRate
1	"JS21"	"fromFilteredE...	[2,4]	[14,16]	[]
2	"JS17"	"fromFilteredE...	[2,4]	[14,16]	[]
3	"JS13"	"fromFilteredE...	[2,4]	[14,16]	[]

	animal	generateH	epochsSelected		samplingRate
4	"ER1"	"fromFilteredE...	[2,4]	[14,16]	[]
5	"JS14"	"fromFilteredE...	[2,4]	[14,16]	[]
6	"ZT2"	"fromFilteredE...	[2,4]	[14,16]	[]
7	"JS17"	"fromFilteredE...	[2,4,6]	[12,14,16]	[]
8	"JS14"	"fromFilteredE...	[2,4,6]	[12,14,16]	[]
9	"JS21"	"fromFilteredE...	[2,4,6]	[12,14,16]	[]
10	"ER1"	"fromFilteredE...	[2,4,6]	[12,14,16]	[]
11	"JS13"	"fromFilteredE...	[2,4,6]	[12,14,16]	[]
12	"ZT2"	"fromFilteredE...	[2,4,6]	[12,14,16]	[]

```
if multi_epoch
    keys = [];
    for i = 1:height(matching_runs)
        if numel(matching_runs(i,:).epochsSelected{1} ) ==3 % change this to
3 for 3 early and 3 late
            keys = [keys, matching_runs(i,:).hash];
        end
    end
else
    keys = matching_runs.hash;
end
```

```
% Load up combined pattern data
if ispc
    [Patterns, otherData] = query.combinePatterns(keys);
else
    [Patterns, otherData] = query.pattern.tmpLoadAndCombine(keys);
end
```

```
Warning: Name is nonexistent or not a directory: C:\Users\BrainMaker\MATLAB
Drive\Shared\hash_multiEpoch
Loading keys:  000%  |                                    | 0/6 [00:00:00<Inf:NaN:NaN, Inf it/s]
```

by now, Patterns is

(nAnimal * nMethods * nPartition * nDirection * nPattern+Control)

Setup constants and aliases

```
[nAnimal, nMethods, nPartition, ~, nResult] = size(Patterns)
```

```
nAnimal = 6
nMethods = 2
nPartition = 50
nResult = 6
```

```
nPatterns = nResult/2;
```

```
for i = 1:nAnimal
    for j = 1:nMethods
        for p = 1:nPartition
            for d = 1:2
                for n = 1:nResult
                    Patterns(i,j,p,d,n).animal =  otherData{i}.Option.animal;
                end
            end
        end
    end
end
```

```
nSource = zeros(1,nAnimal);
nTarget = zeros(1,nAnimal);
numDimsUsedForPrediction = cell(1,nAnimal);
for a = 1:nAnimal
    nSource(a) = size(Patterns(a,1,1,1,1).X_source,1);
    nTarget(a) = size(Patterns(a,1,1,1,1).X_target,1);
    numDimsUsedForPrediction{a} = 1:min(nSource(a), nTarget(a));
end
```

```
Option = otherData{1}.Option;
if Option.sourceArea == "CA1"
    source = "hpc";
    target = "pfc";
    hpc = 1;
    pfc = 2;
else
    source = "pfc";
    target = "hpc";
    hpc = 2;
    pfc = 1;
end
```

The different animals loaded will actually collapse into the partition

dimenion, segregated by the genH methods

(nMethods * nPartiton * nDirection * nPatterns)

```
tempPatterns = permute(Patterns, [2,1,3,4,5]);
newSize = size(tempPatterns);
newSize = [newSize(1), prod(newSize(2:3)), newSize(4:end)];
Patterns_AllAnimals = reshape(tempPatterns, newSize);

nAnimalPartition = nAnimal * nPartition;
patternnames = ["theta", "delta", "ripples"];
```

```
T = query.getPatternTable(Patterns_AllAnimals);
```

```
Creating pattern tab:  000%  |                              | 0/7200 [00:00:00<Inf:NaN:NaN, Inf it
```


Figure 2A: Cofiring in source and target

Figure 2B: Prediction Performance

Figure 4A/B: Results from rank regress

Figure 4C: How optimal number of predicitive dimensions compare between hpc and pfc

Figure 6: removing predicitive dimensions from both sources

Figure 7: Predictive performance from dominant dimensions

Figure 2

A: How pairs of neuron in source/target co-fire

This is method insensitive. Just lump over animals

```
% calculate co-firing across all animals
cofiring
```

```
Processing:  000%  |                                  | 0/3 [00:00:00<Inf:NaN:NaN, Inf it
```

The co-firing of hpc and pfc neurons during different activity patterns, with average plotted

```
plotCofiring
```

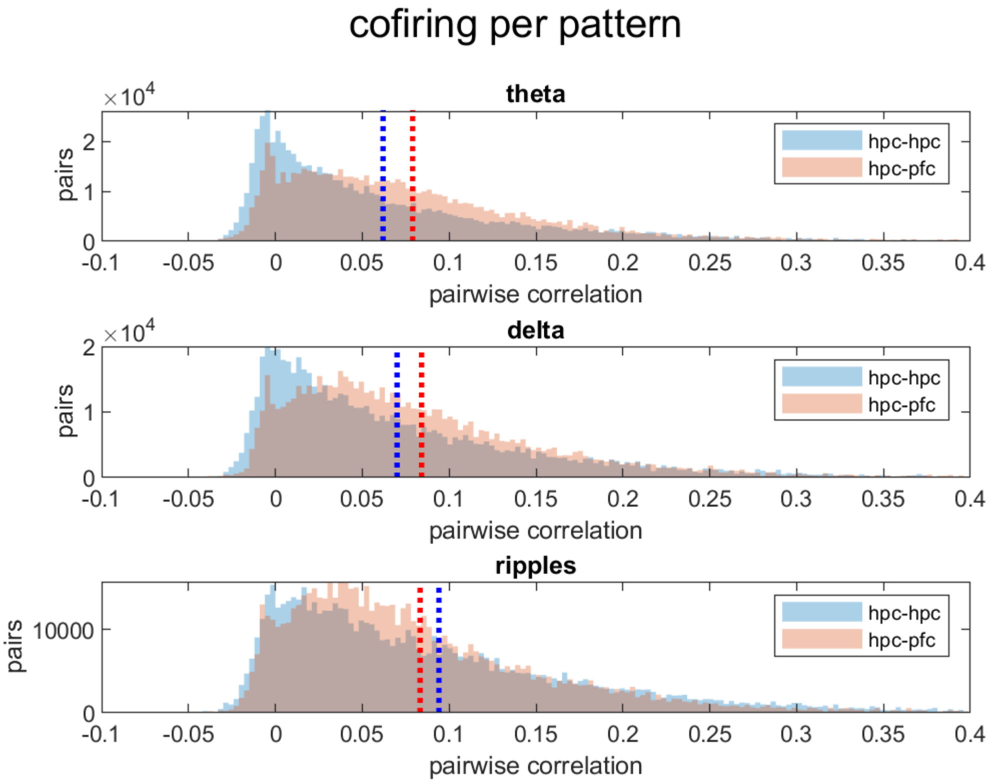

the difference in cofiring between hpc-hpc pairs and hpc-pfc pairs are significant for all the activity patterns

```
% How about just put all the patterns together?
fig 'Overall directional correlation'
subplot(2,1,1)
ax1 = nexttile;
hist_withpfc = histogram(all_pairs_withhpc)
```

```
hist_withpfc =
  Histogram with properties:

             Data: [1×346800 double]
           Values: [1×160 double]
          NumBins: 160
         BinEdges: [1×161 double]
         BinWidth: 0.0050
        BinLimits: [-0.0800 0.7200]
    Normalization: 'count'
        FaceColor: 'auto'
        EdgeColor: [0 0 0]

  Show all properties
```

```
ylabel("Pairs")
title ("HPC-HPC")
example_mean_corr_withhpc = mean_corrwithhpc(1);
example_std_corr_withhpc = std_corrwithhpc(1);

hold on
```

```
lineObject=line([example_mean_corr_withhpc,example_mean_corr_withhpc],[0
max(hist_withpfc.Values)]);
lineObject.LineStyle = ':'; % Make line dotted
lineObject.LineWidth = 2;  % Thicken the line
lineObject.Color = 'black'; % Color it black
```

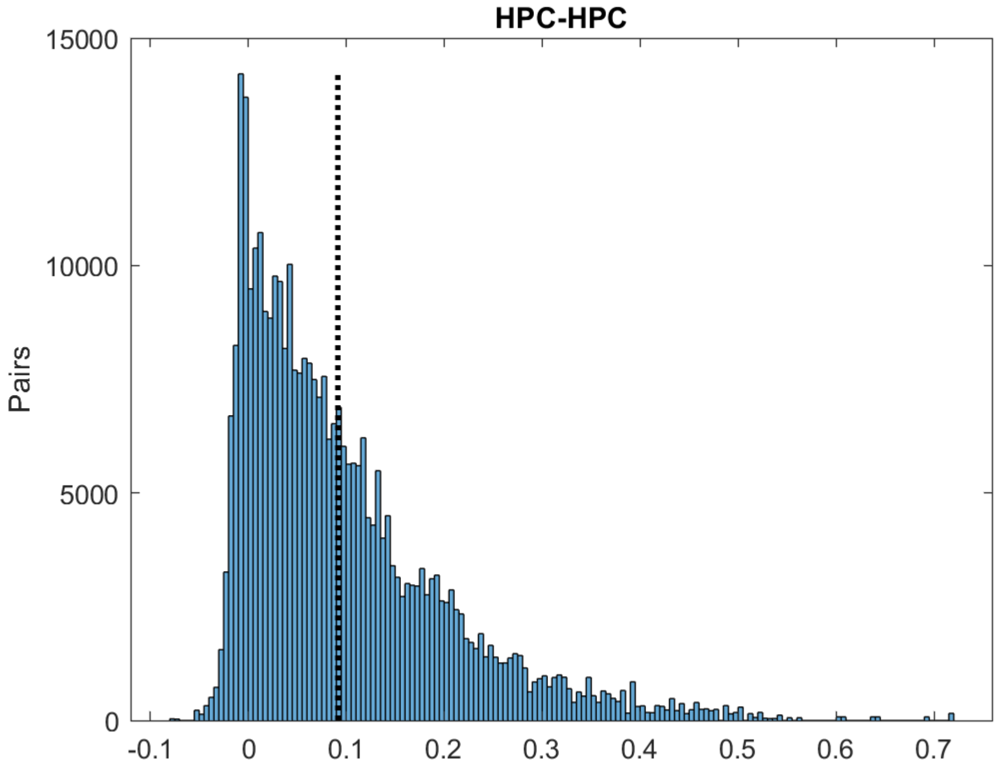


```
subplot(2,1,2)
ax2 = nexttile;
hist_hp = histogram(all_pairs_withpfc)
```

```
hist_hp =
  Histogram with properties:

             Data: [1×346800 double]
           Values: [1×160 double]
          NumBins: 160
         BinEdges: [1×161 double]
         BinWidth: 0.0050
        BinLimits: [-0.0800 0.7200]
    Normalization: 'count'
        FaceColor: 'auto'
        EdgeColor: [0 0 0]

  Show all properties
```

```
ylabel("Pairs")
title ("HPC-PFC")
example_mean_corr_withpfc = mean_corrwithpfc(1);
example_std_corr_withpfc = std_corrwithpfc(1);
```

```
lineObject=line([example_mean_corr_withpfc,example_mean_corr_withpfc],[0
max(hist_hp.Values)]);
lineObject.LineStyle = ':'; % Make line dotted
lineObject.LineWidth = 2;  % Thicken the line
lineObject.Color = 'black'; % Color it black
xlabel("Pairwise correlation")
linkaxes([ax1,ax2],'x');
```

```
Warning: Excluding ColorBars, Legends and non-axes
```

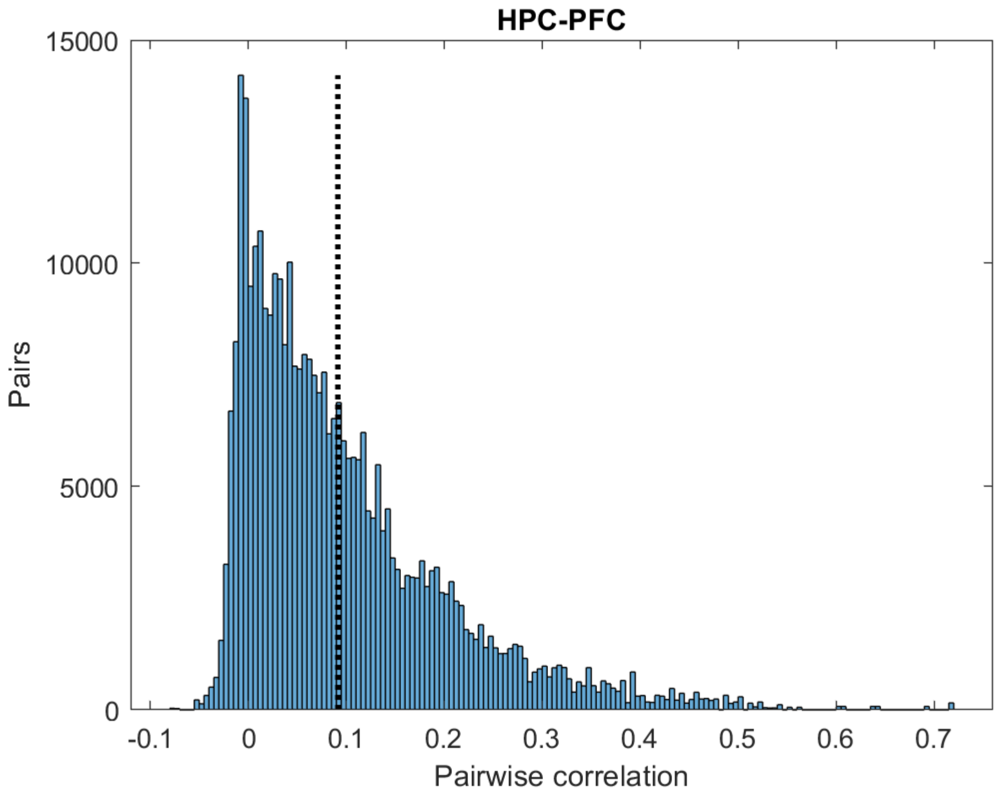


```
[h_corrdiff,p_corrdiff] = kstest2(all_pairs_withpfc,all_pairs_withhpc);
```

cofiring = 1×6 table

	Var1	Var2	Var3	Var4	h_corrdiff	p_corrdiff
1	0.0547	0.0547	0.0575	0.0575	1	1.8130e-131

```
% print stats
formatSpec1 = "%s: %0.3f±%0.3f";

sprintf(formatSpec1,Patterns(1,1).directionality,mean(mean_corrwithhpc),mean(
std_corrwithhpc))
```

```
ans =
"pfc-pfc: 0.055±0.055"
```

```
sprintf(formatSpec1,Patterns(2,1).directionality,mean(mean_corrwithpfc),mean(
std_corrwithpfc))
```

```
ans =
"pfc-pfc: 0.057±0.057"
```

```
disp(p_corrdiff)
```

```
1.8130e-131
```

"These weak correlations indicate that only a small fraction of a neuron's response variability can be explained by another individual neuron"

B: Explained Variance

```
regionalVarExplained;
```

```
clf;plotVarExplained;
```

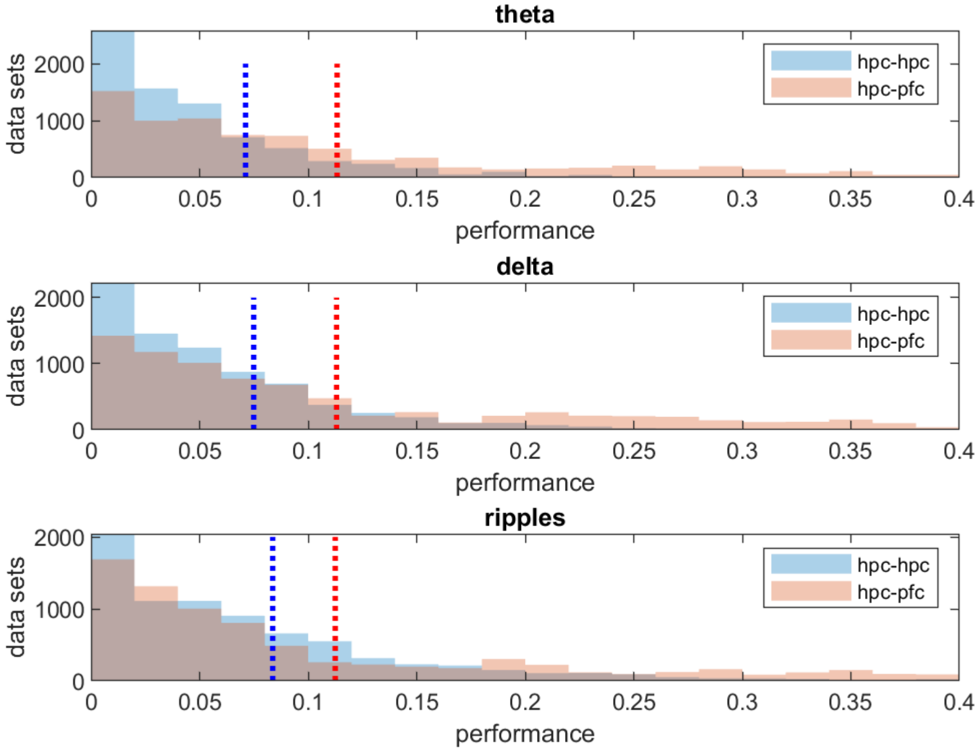

Predicition Performance

```

```

```
[h_hpc, p_hpc] = kstest2(meanpred_hpc, mean_withhpccorr_pattern)
```

```
Error using kstest2 (line 97)
The samples X1 and X2 must be vectors.
```

```
[h_pfc, p_pfc] = kstest2(meanpred_pfc, mean_withpfccorr_pattern)
```

```
mean_withhpc = mean(r_square_withhpc(intersect(~isinf(r_square_withhpc),
~isnan(r_square_withhpc))));
```

```
Unrecognized function or variable 'r_square_withhpc'.
```

```
std_withhpc  = std(r_square_withhpc(intersect(~isinf(r_square_withhpc),
~isnan(r_square_withhpc))));

mean_withpfc = mean(r_square_withpfc(intersect(~isinf(r_square_withpfc),
~isnan(r_square_withpfc))));
std_withpfc = std(r_square_withpfc(intersect(~isinf(r_square_withpfc),
~isnan(r_square_withpfc))));

subplot(2,1,1)
ax1 = nexttile;
hist_withpfc = histogram(r_square_withhpc,25)
ylabel("Data Sets")
title ("source predicting HPC targets")
lineObject=line([mean_withhpc,mean_withhpc],[0 max(hist_withpfc.Values)]);
lineObject.LineStyle = ':'; % Make line dotted
lineObject.LineWidth = 2;  % Thicken the line
lineObject.Color = 'black'; % Color it black

lineObject2 = line([median_singlehh,median_singlehh],[0
max(hist_withpfc.Values)]);
lineObject2.LineWidth = 3;
lineObject2.Color = 'blue';
```

```


subplot(2,1,2)
ax2 = nexttile;
hist_hp = histogram(r_square_withpfc,25)
```

```
hist_hp = 
  Histogram with properties:

             Data: [1×720 double]
           Values: [160 78 54 58 58 49 30 21 14 17 11 6 17 20 10 15 16 9 2 4 7 3 5 4 2]
          NumBins: 25
         BinEdges: [0 0.0173 0.0346 0.0519 0.0692 0.0865 0.1038 0.1211 0.1384 0.1557 0.1730 0.1903 0.2076
         BinWidth: 0.0173
        BinLimits: [0 0.4325]
    Normalization: 'count'
        FaceColor: 'auto'
        EdgeColor: [0 0 0]
```

Show all properties

```
% prediction performance from hpc to pfc cells
ylabel("Data Sets")
title ("source predicting PFC targets")
xlabel("Performance")
linkaxes([ax1,ax2],'x');
```

Warning: Excluding ColorBars, Legends and non-axes

```
lineObject=line([mean_withpfc,mean_withpfc],[0 max(hist_hp.Values)]);
lineObject.LineStyle = ':'; % Make line dotted
lineObject.LineWidth = 2;  % Thicken the line
lineObject.Color = 'black'; % Color it black

lineObject2 = line([median_singlehp,median_singlehp],[0
max(hist_hp.Values)]);
lineObject2.LineWidth = 3;  % Thicken the line
lineObject2.Color = 'blue'; % Color it black
```

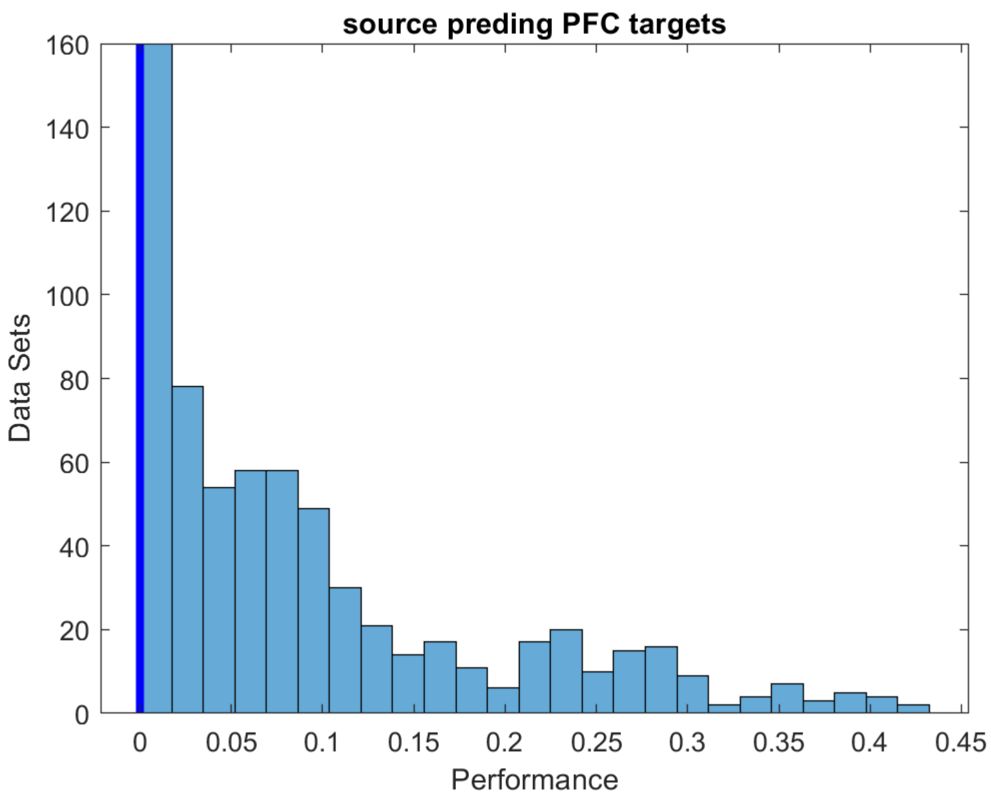


```
figure(601)
clf
for i = 1:nPatterns
    subplot(3,1,i)
    plot(patternPerformance_pfc(i,:));
```

```
    hold on
    plot(patternPerformance_hpc(i,:));

    legend("pfc","hpc")
    % how to interpret nans
end
```

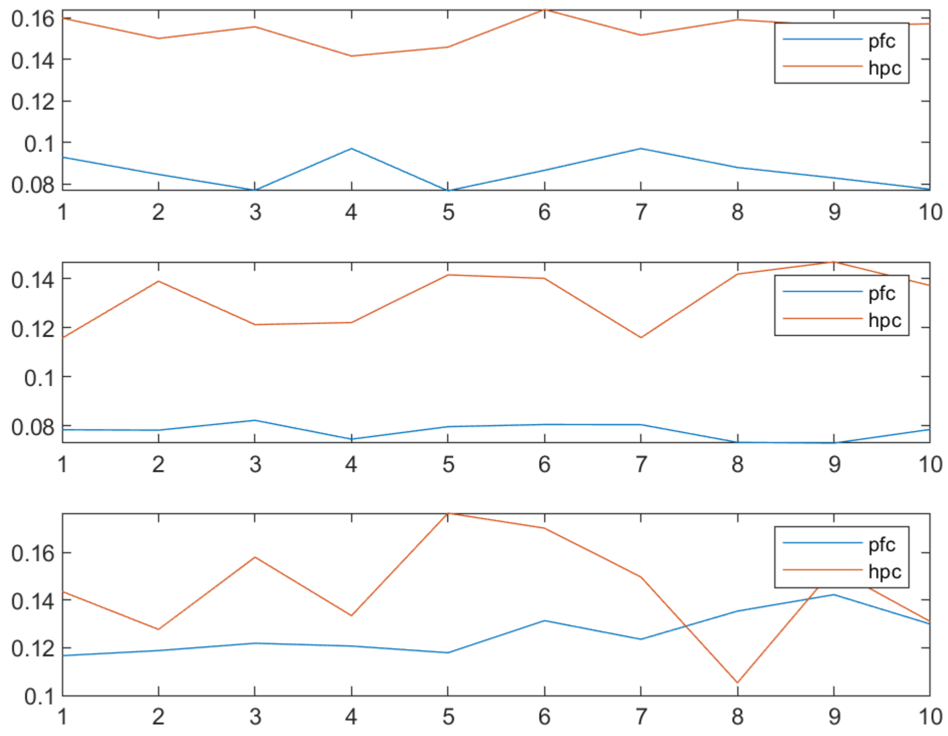


```
Predictiontable = 1×4 table
```

	mean_hh	std_hh	mean_hp	std_hp
1	0.0190	0	0.0263	0

```
predictionRow = 1×2 table
```

	Predictiontable	key
1	1×4 table	"5295fea"

```
% print stats
formatSpec1 = "%s: %0.3f±%0.3f";
disp("Prediction Performance on Average")
```

```
Prediction Performance on Average
```

```
sprintf(formatSpec1,Patterns(1,1).directionality,mean_withhpc,std_withhpc)
```

```
ans = 
"hpc-hpc: 0.091±0.102"
```

```
sprintf(formatSpec1,Patterns(2,1).directionality,mean_withpfc,std_withpfc)
```

```
ans =
"hpc-hpc: NaN±NaN"
```

```
if useSinglePrediction
    disp('single source prediction median')
    formatSpec2 = "%s: %0.5e";
    sprintf(formatSpec2,Patterns(1,1).directionality,median_singlehh)
    sprintf(formatSpec2,Patterns(1,2).directionality,median_singlehp)
end
```

```
single source prediction median
ans =
"hpc-hpc: -5.32686e-06"
ans =
"hpc-pfc: 4.57062e-04"
```

Figure 4

```
withPredDims;
```

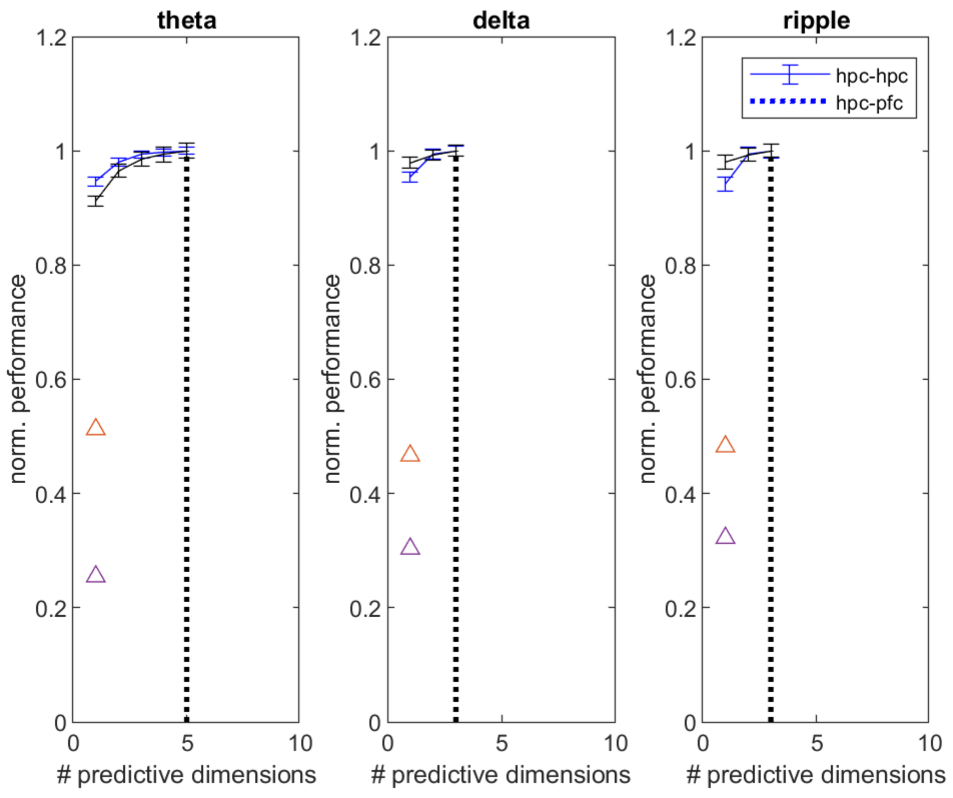


```
% histogram of all predicition perf
hpc_theta = T.generateH == 'fromFilteredEEG  fromRipTimes'...
    & T.directionality == "hpc-hpc" & T.patternType == "theta";
hpc_delta = T.generateH == 'fromFilteredEEG  fromRipTimes'...
    & T.directionality == "hpc-hpc" & T.patternType == "delta";
hpc_ripple = T.generateH == 'fromFilteredEEG  fromRipTimes'...
```

```
    & T.directionality == "hpc-hpc" & T.patternType == "ripple";


pfc_theta = T.generateH == 'fromFilteredEEG  fromRipTimes'...
    & T.directionality == "hpc-pfc" & T.patternType == "theta";
pfc_delta = T.generateH == 'fromFilteredEEG  fromRipTimes'...
    & T.directionality == "hpc-pfc" & T.patternType == "delta";
pfc_ripple = T.generateH == 'fromFilteredEEG  fromRipTimes'...
    & T.directionality == "hpc-pfc" & T.patternType == "ripple";

hpc_pred =
{T(hpc_theta,:).full_model_performance,T(hpc_delta,:).full_model_performance,
T(hpc_ripple,:).full_model_performance};
pfc_pred =
{T(pfc_theta,:).full_model_performance,T(pfc_delta,:).full_model_performance,
T(pfc_ripple,:).full_model_performance};



h_corrdiff = zeros(1,nPatterns);
p_corrdiff = zeros(1,nPatterns);
for i = 1:3
    subplot(3,1,i);
    hold off;
    predperf_hh = histogram(hpc_pred{i});
    set(predperf_hh, 'EdgeColor', 'none', 'FaceAlpha', 0.33);
    hold on
    predperf_hp = histogram(pfc_pred{i});
    set(predperf_hp, 'EdgeColor', 'none', 'FaceAlpha', 0.33);

    title(patternnames(i))
    ylabel("data sets")

    hold on
    avg_hh=line([mean(hpc_pred{i}),mean(hpc_pred{i})],[0
max(predperf_hh.Values)]);
    avg_hh.LineStyle = ':'; % Make line dotted
    avg_hh.LineWidth = 2;  % Thicken the line
    avg_hh.Color = 'blue';
    avg.hh.DisplayName = "hpc-hpc corfiring mean";
    %
    hold on
    avg_hp=line([mean(pfc_pred{i}),mean(pfc_pred{i})],[0
max(predperf_hp.Values)]);
    avg_hp.LineStyle = ':'; % Make line dotted
    avg_hp.LineWidth = 2;  % Thicken the line
    avg_hp.Color = 'red';
    avg.hh.DisplayName = "hpc-pfc corfiring mean";

    if source == "hpc"
```

```
        legend("hpc-hpc","hpc-pfc")
    else
        legend("pfc-hpc","pfc-pfc")
    end

    xlabel("predicition performance")
    [h_corrdiff(i),p_corrdiff(i)] = ttest2(hpc_pred{i}(~isnan(hpc_pred{i})),
pfc_pred{i}(~isnan(pfc_pred{i})));
    [h_pred(i),p_pred(i)] = kstest2(hpc_pred{i}(~isnan(hpc_pred{i})),
pfc_pred{i}(~isnan(pfc_pred{i})));
    xlim([0,0.75])

end
```

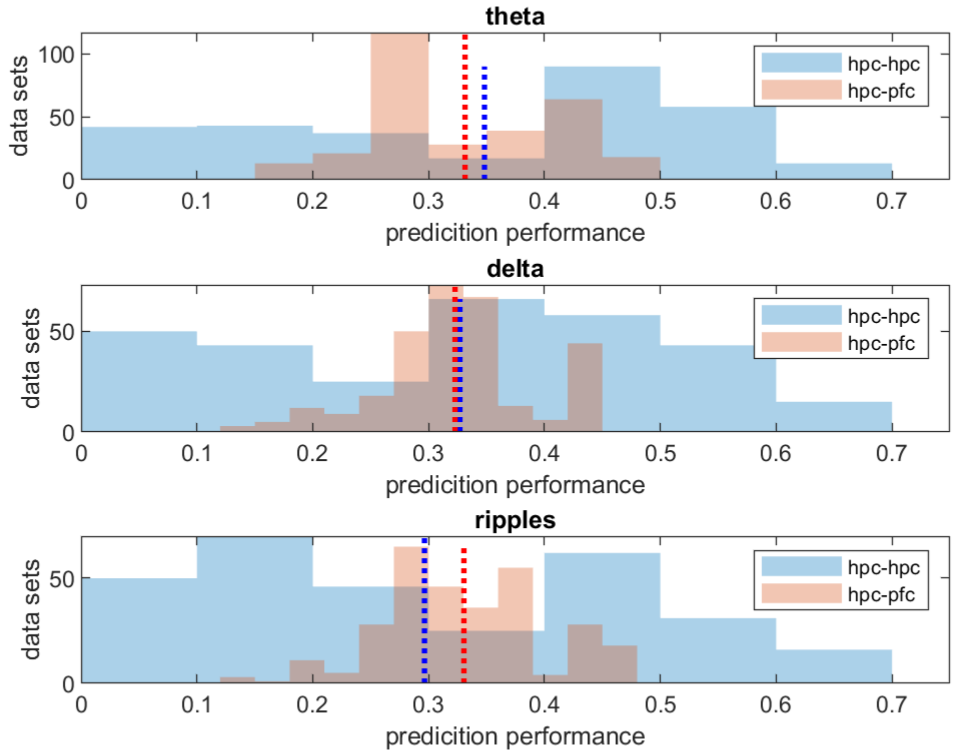

Figure 5 - Showing the complexity not due to the other region being more complex

A: (difference in qOpt)

```
temp1 = zeros(3,nPartitions);
```

```
temp2 = zeros(3,nPartitions);
temp3 = zeros(3,nPartitions);

for j = 1:nPartitions
    for k = 1:nPatterns
        temp2(k,j) = Patterns(j,hpc,k).factorAnalysis.qOpt;
        temp3(k,j) = Patterns(j,pfc,k).factorAnalysis.qOpt;
        temp1(k,:) = temp2(k,:)./temp3(k,:);
    end
end
```

```
Unable to perform assignment because the size of the left side is 1-by-1 and the size of the right
side is 0-by-0.
```

```
%
ratios = mean(temp1,2);
hpcQoptDims = mean(temp2,2);
pfcQoptDims = mean(temp3,2);
```

Figure 6

Pattern Specific

```
removePred
```

```
iPartition:   000%  |                                 | 0/300 [00:00:00<Inf:NaN:NaN, Inf it/s]
```

Remove tuples of (targetArea,pattern) from a given (targetArea, pattern)

```
figure;
g = gramm(...
    'x',rt.dimensionRemoved, ...
    'y', rt.performance, ...
    'color', categorical(rt.removePattern), ...
    'lightness', categorical(rt.sameDirectionLabel),...
    'linestyle', categorical(rt.sameDirectionLabel));
g = g.facet_grid(categorical(rt.basePatternLabel),
categorical(rt.targetArea));
g = g.stat_summary('geom','line');
g = g.stat_summary('geom','point');
g = g.stat_summary('geom','errorbar');
g = g.stat_summary('geom','area');
g.set_text_options("interpreter",'latex');
g = g.set_names('x','dims removed',...
    'y','peformance',...
    'column','Interaction',...
    'row','Pattern', ...
```

```
    'color','Pattern dims removed',...
    'lightness',"Remove Same/Different"+newline+"Pred. Target",...
    'linestyle',"Remove Same/Different"+ newline+"Pred. Target");
g.draw();
warning off; set(g.facet_axes_handles, 'yscale','log'); warning on;
```

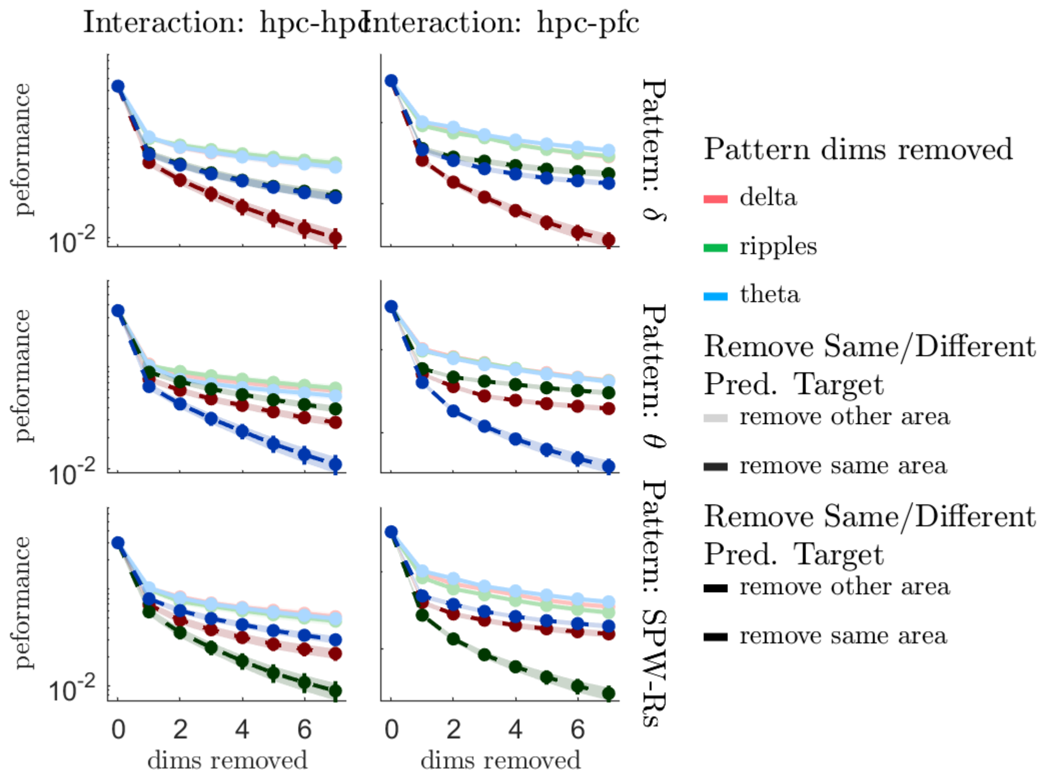


```
Warning: Negative limits ignored
Warning: Negative limits ignored
Warning: Negative limits ignored
Warning: Negative limits ignored
Warning: Negative limits ignored
Warning: Negative limits ignored
Warning: Negative limits ignored
Warning: Negative limits ignored
Warning: Negative limits ignored
Warning: Negative limits ignored
Warning: Negative limits ignored
Warning: Negative limits ignored
```

Remove pattern from same target area

```
figure;
g = gramm(...
    'x',rt.dimensionRemoved, ...
    'y', rt.performance, ...
    'color', categorical(rt.removePattern), ...
    'subset',rt.sameDirection);
g = g.facet_grid(categorical(rt.basePatternLabel),
categorical(rt.targetArea));
g = g.stat_summary('geom','line');
```

```
g = g.stat_summary('geom','point');
g = g.stat_summary('geom','errorbar');
g = g.stat_summary('geom','area');
g.set_text_options("interpreter",'latex');
g=g.set_title("Removing dimensions" + newline + "(of similar target area
only)");
g = g.set_names('x','dims removed',...
    'y','peformance',...
    'column','Interaction',...
    'row','Pattern', ...
    'color','Pattern dims removed');
g.draw();
warning off; set(g.facet_axes_handles, 'yscale','log'); warning on;
```

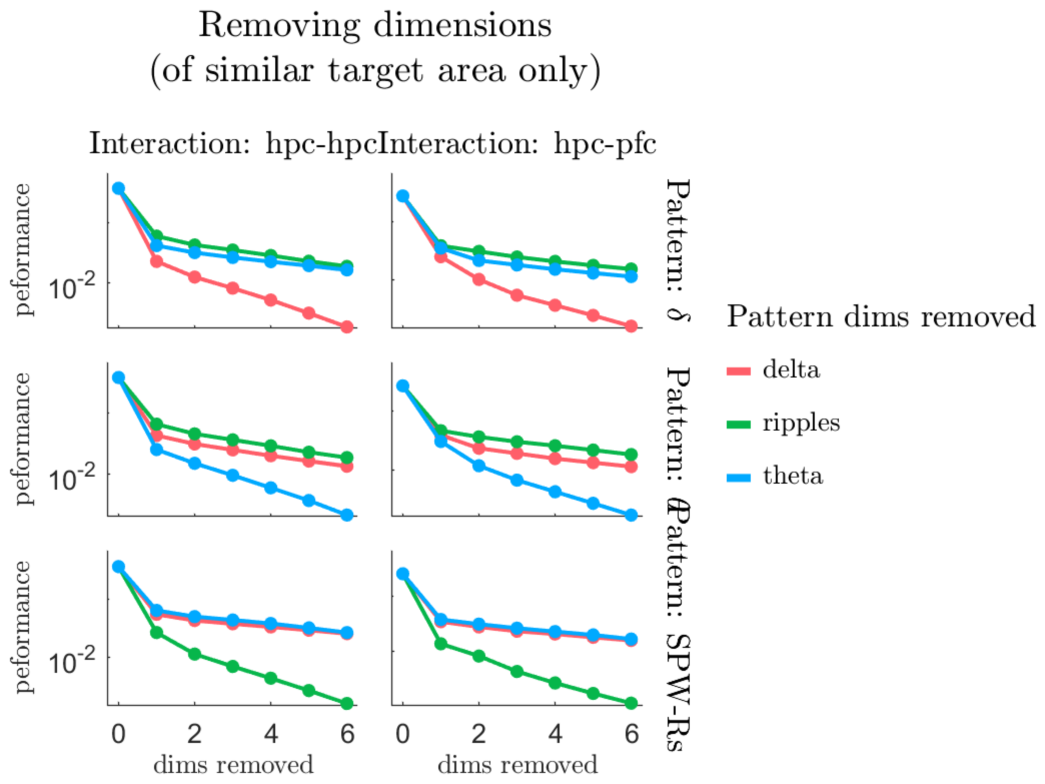


```
Warning: Negative limits ignored
Warning: Negative limits ignored
Warning: Negative limits ignored
Warning: Negative limits ignored
Warning: Negative limits ignored
Warning: Negative limits ignored
Warning: Negative limits ignored
Warning: Negative limits ignored
Warning: Negative limits ignored
Warning: Negative limits ignored
Warning: Negative limits ignored
Warning: Negative limits ignored
```

```
figure;
g = gramm(...
```

```
    'x',rt.dimensionRemoved, ...
    'y', rt.performance, ...
    'color', categorical(rt.removePattern), ...
    'subset',~rt.sameDirection);
g = g.facet_grid(categorical(rt.basePatternLabel),
categorical(rt.targetArea));
g = g.stat_summary('geom','line');
g = g.stat_summary('geom','point');
g = g.stat_summary('geom','errorbar');
g = g.stat_summary('geom','area');
g = g.set_color_options('lightness',200);
g.set_text_options("interpreter",'latex');
g = g.set_names('x','dims removed',...
    'y','peformance',...
    'column','Interaction',...
    'row','Pattern', ...
    'color','Pattern dims removed');
g=g.set_title('Removing different only')
```

```
g = 
  gramm with properties:

      legend_axe_handle: []
       title_axe_handle: []
     facet_axes_handles: []
                results: [1×1 struct]
```

```
g.draw();
warning off; set(g.facet_axes_handles, 'yscale','log'); warning on;
```

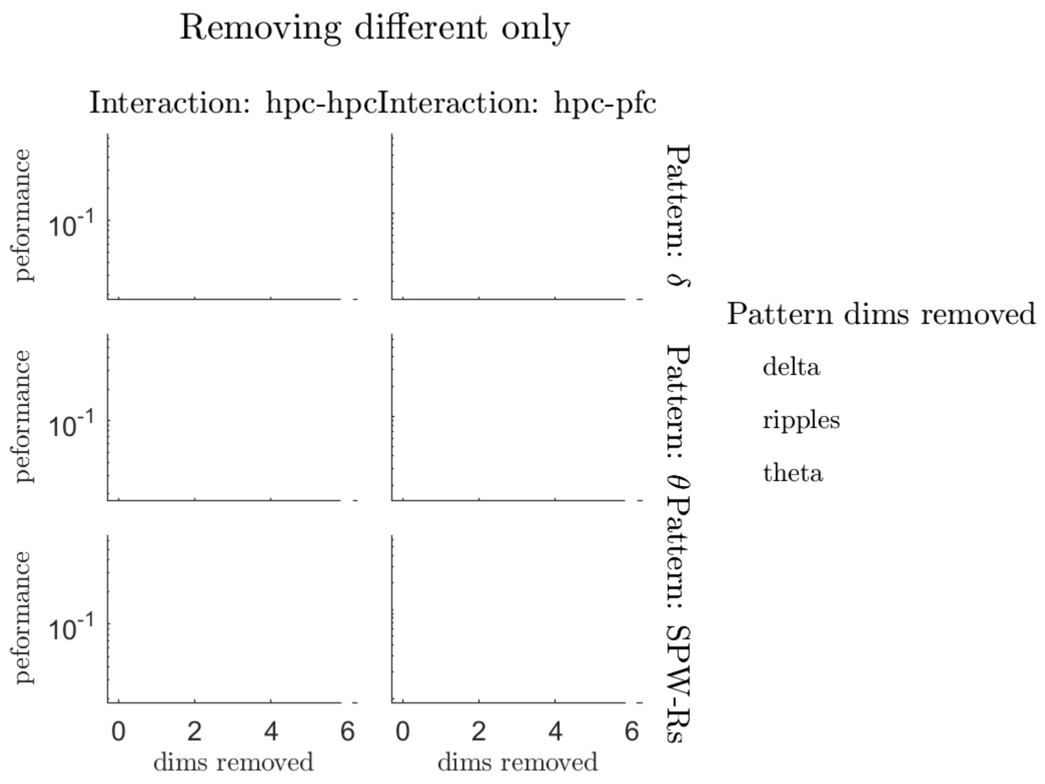


```
Warning: Negative limits ignored
Warning: Negative limits ignored
Warning: Negative limits ignored
Warning: Negative limits ignored
Warning: Negative limits ignored
Warning: Negative limits ignored
Warning: Negative limits ignored
Warning: Negative limits ignored
Warning: Negative limits ignored
Warning: Negative limits ignored
Warning: Negative limits ignored
Warning: Negative limits ignored
```

Overall target removal

```
figure;
g = gramm(...
    'x',rt.dimensionRemoved, ...
    'marker', rt.targetArea,...
    'y', rt.performance, ...
    'color', categorical(rt.sameDirectionLabel),'subset',~rt.sameDirection);
g = g.facet_grid([],categorical(rt.targetArea));
g = g.stat_summary('geom','line');
g = g.stat_summary('geom','point');
g = g.stat_summary('geom','errorbar');
g = g.stat_summary('geom','area');
g = g.set_color_options('chroma',0,'lightness',30);
g.set_text_options("interpreter",'latex');
g = g.set_names('x','dims removed',...
```

```
    'y','peformance',...
    'column','Interaction',...
    'row','Pattern', ...
    'color','Brain area removed');
snapnow;
g=g.update('subset',rt.sameDirection);
```

```
Check for missing argument or incorrect argument data type in call to function 'isnan'.

Error in unique_and_sort (line 29)
        y(isnan(y)) = []; % remove all nans

Error in gramm/draw (line 217)
uni_marker=unique_and_sort(temp_aes.marker,obj.order_options.marker);

Error in gramm/update (line 20)
    draw(obj);
```

```
%g = g.facet_grid([],categorical(rt.targetArea));
g = g.stat_summary('geom','line');
g = g.stat_summary('geom','point');
g = g.stat_summary('geom','errorbar');
g = g.stat_summary('geom','area');
g = g.set_color_options(); % Restore default color
g.draw();
warning off; set(g.facet_axes_handles, 'yscale','log'); warning on;
```

Now let's see how similar those curves are in dimension reduced space

```
unstack(rt, '');
```

Figure 7

A: Dominant dimension predicitions

```
numUsedForPrediction = min(nTarget,nSource);
% make the averaged version
curr_cvLoss = cell(10,2,3);
curr_qOptDim = cell(10,2,3);
for p = 1:10
    for i = 1:nPatterns
        for j = 1:2
            if ~Patterns(p,j,i).singularWarning
                curr_cvLoss{p,j,i} = Patterns(p,j,i).factorAnalysis.cvLoss;
                curr_qOptDim{p,j,i} =
Patterns(p,j,i).factorAnalysis.optDimFactorRegress;
            end
        end
    end
end
```

```
figure(750)
clf
full_model_performance = [];

for i = 1:nPatterns
    for j = 1:2
        subplot(3,2,2*(i-1)+j)

        full_model =
plots.plotPredictiveDimensions(numUsedForPrediction,curr_cvLoss(:,j,i),'optDi
m',curr_qOptDim, ...
            "mode", "fa");

        full_model_performance = [full_model_performance, full_model];
        xlim([0,12.5])
        hold on

        plot(1, full_model,'^');
        ylim([0, full_model+0.1])
        if j == 1
            ax1 = gca;
        else
            ax2 = gca;
        end
        title([Patterns(p,j,i).name Patterns(p,j,i).directionality])
    end
    linkaxes([ax1,ax2],'y')

end
```

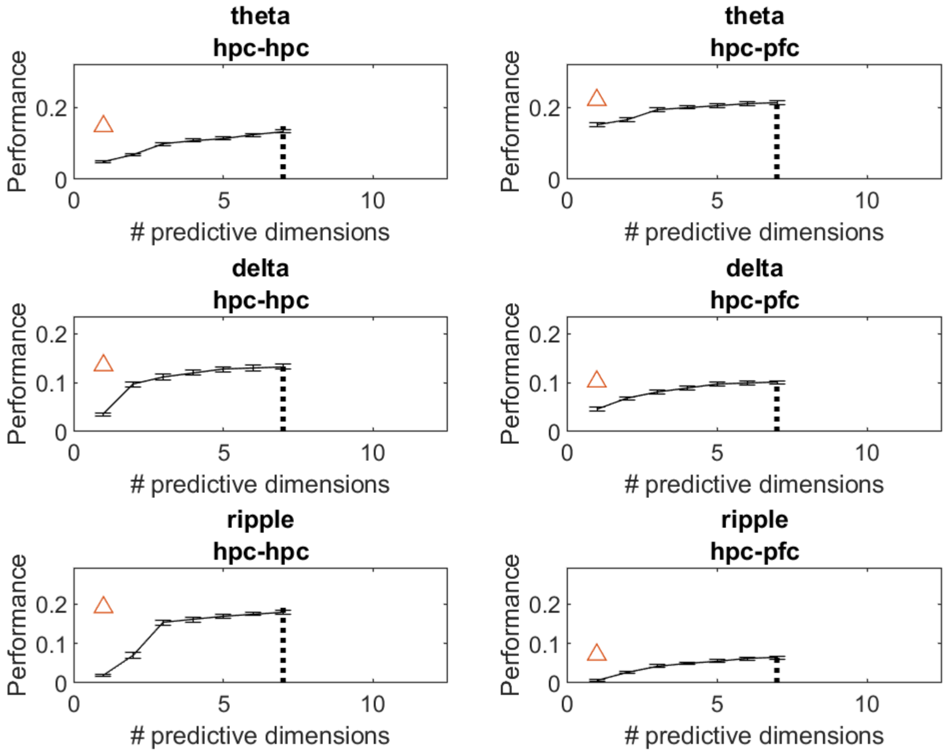
theta
hpc-hpc
Performance
0.2
0
0
5
10
predictive dimensions
theta
hpc-pfc
Performance
0.2
0
0
5
10
predictive dimensions
delta
hpc-hpc
Performance
0.2
0.1
0
0
5
10
predictive dimensions
delta
hpc-pfc
Performance
0.2
0.1
0
0
5
10
predictive dimensions
ripple
hpc-hpc
Performance
0.2
0.1
0
0
5
10
predictive dimensions
ripple
hpc-pfc
Performance
0.2
0.1
0
0
5
10
predictive dimensions
