# Supplementary figures and images for: Hippocampal–Prefrontal Communication Subspaces Align with Behavioral and Network Patterns in a Spatial Memory Task
Source: eNeuro. 2025 Sep 18;12(9):ENEURO.0336-24.2025. doi: 10.1523/ENEURO.0336-24.2025 (PMC12463551; doi:10.1523/ENEURO.0336-24.2025)

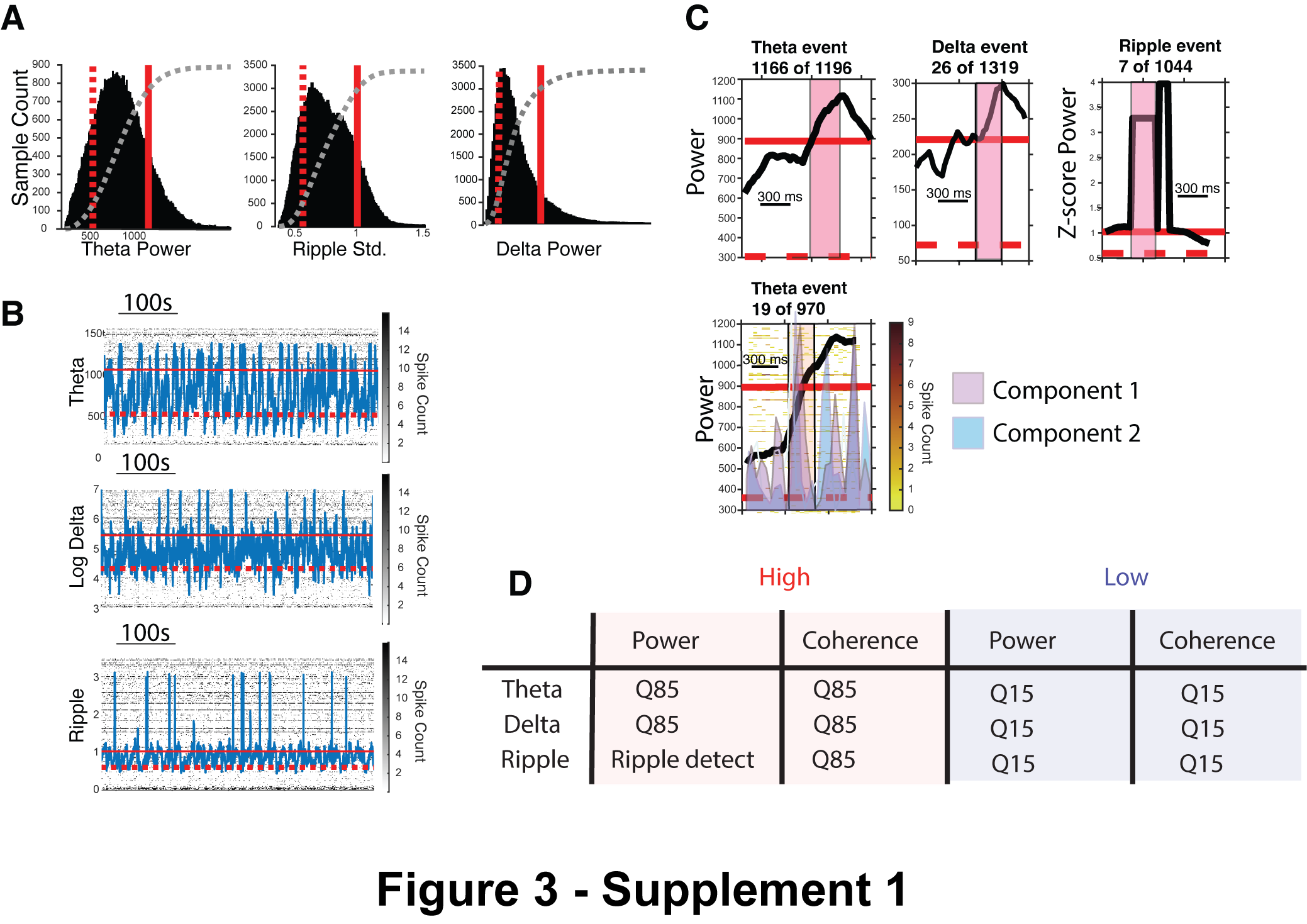

Supplement: Figure 3-1 — Windowing of high and low network pattern events. Download Figure 3-1, TIF file. [file eneuro-12-ENEURO.0336-24.2025-s004.tif]

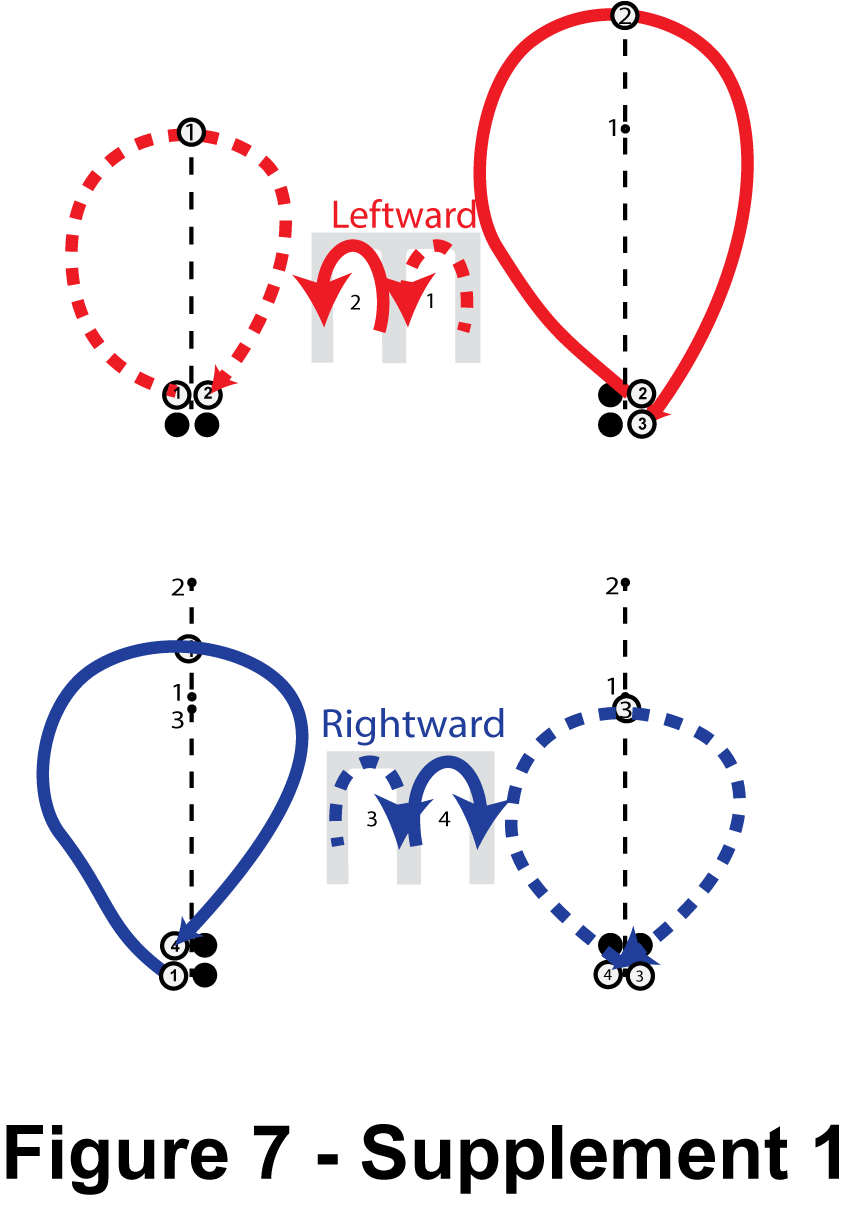

Supplement: Figure 7-1 — Attractor Hypothesis. The four trajectories are schematized. Inbound is shown in dashed line style, outbound in solid line style, leftward is red, and rightward is blue. The trajectories form neighboring rings—all proceeding clockwise (Figure 7). Different trajectories are labeled 1-4 near their respective choice point schematics. Active start/end and choice points are shown in white. Though we have not explored the initial positions, we hypothesize that neighboring ring attractors could distinguish trajectory class and may terminate near the start of the upcoming trajectory. In this manner, such CA1-PFC coactivity could implement a form of task logic in the communication space. Download Figure 7-1, TIF file. [file eneuro-12-ENEURO.0336-24.2025-s005.tif]
